# Development of 3D-printed universal adapter in enhancing retinal imaging accessibility

**DOI:** 10.1186/s41205-024-00231-0

**Published:** 2024-07-19

**Authors:** Aisya Amelia Abdul Latip, Kuryati Kipli, Abang Mohammad Nizam Abang Kamaruddin, Rohana Sapawi, Kasumawati Lias, Muhammad Arif Jalil, Khairul Fikri Tamrin, Nurul Mirza Afiqah Tajudin, Han Yi Ong, Muhammad Hamdi Mahmood, Suriati Khartini Jali, Siti Kudnie Sahari, Dayang Azra Awang Mat, Lik Thai Lim

**Affiliations:** 1 Department of Electrical and Electronics Engineering, Universiti Malaysia Sarawak (UNIMAS), Kota Samarahan, Sarawak, 94300 Malaysia; 2 Department of Mechanical and Manufacturing Engineering, Universiti Malaysia Sarawak (UNIMAS), Kota Samarahan, Sarawak, 94300 Malaysia; 3 Department of Physics, Faculty of Science, Universiti Teknologi Malaysia (UTM), Skudai, Johor, 81310 Malaysia; 4 Department of Clinical Science, Faculty of Medicine and Health Sciences (FMHS), Universiti Malaysia Sarawak (UNIMAS), Kota Samarahan, 94300 Malaysia; 5 Department of Basic Medical Sciences, Faculty of Medicine and Health Sciences (FMHS), Universiti Malaysia Sarawak (UNIMAS), 94300 Kota Samarahan, Malaysia; 6 Faculty of Computer Science and Information Technology, Universiti Malaysia Sarawak (UNIMAS), Kota Samarahan, Sarawak, 94300 Malaysia; 7 Department of Ophthalmology, Faculty of Medicine and Health Sciences (FMHS), Universiti Malaysia Sarawak (UNIMAS), Kota Samarahan, 94300 Malaysia

**Keywords:** Smartphone, Adapter, Universal, Ophthalmoscope

## Abstract

**Background:**

The revolutionary technology of smartphone-based retinal imaging has been consistently improving over the years. Smartphone-based retinal image acquisition devices are designed to be portable, easy to use, and cost-efficient, which enables eye care to be more widely accessible especially in geographically remote areas. This enables early disease detection for those who are in low- and middle- income population or just in general has very limited access to eye care. This study investigates the limitation of smartphone compatibility of existing smartphone-based retinal image acquisition devices. Additionally, this study aims to propose a universal adapter design that is usable with an existing smartphone-based retinal image acquisition device known as the PanOptic ophthalmoscope. This study also aims to simulate the reliability, validity, and performance overall of the developed prototype.

**Methods:**

A literature review has been conducted that identifies the limitation of smartphone compatibility among existing smartphone-based retinal image acquisition devices. Designing and modeling of proposed adapter were performed using the software AutoCAD 3D. For the proposed performance evaluation, finite element analysis (FEA) in the software Autodesk Inventor and 5-point scale method were demonstrated.

**Results:**

Published studies demonstrate that most of the existing smartphone-based retinal imaging devices have compatibility limited to specific older smartphone models. This highlights the benefit of a universal adapter in broadening the usability of existing smartphone-based retinal image acquisition devices. A functional universal adapter design has been developed that demonstrates its compatibility with a variety of smartphones regardless of the smartphone dimension or the position of the smartphone’s camera lens. The proposed performance evaluation method generates an efficient stress analysis of the proposed adapter design. The end-user survey results show a positive overall performance of the developed universal adapter. However, a significant difference between the expert's views on the developed adapter and the quality of images is observed.

**Conclusion:**

The compatibility of existing smartphone-based retinal imaging devices is still mostly limited to specific smartphone models. Besides this, the concept of a universal and suitable adapter for retinal imaging using the PanOptic ophthalmoscope was presented and validated in this paper. This work provides a platform for future development of smartphone-based ophthalmoscope that is universal.

**Supplementary Information:**

The online version contains supplementary material available at 10.1186/s41205-024-00231-0.

## Background

Smartphones in general offer a variety of applications in medicine, including for example mobile access to patient relevant information, medical database, patient and health care professional education, and act as a medical diagnostic tool [1–3]. With the emerging ophthalmology technology in smartphones, eye care has become more accessible. Recent development in smartphone-based fundus photography has allowed a cost effective and efficient approach for screening purposes for a variety of diseases, for example diabetic retinopathy. Current smartphone-based retinal image acquisition device is being developed with the intention of being able to provide early diseases detection for those who are in low- and middle- income populations or in general have limited access to ophthalmic examination [4, 5]. For instance, a study by Rajalakshmi et al. [6] stated that developing an automated method involving a computer-based analysis of fundus images would significantly increase the efficiency and reduce the workload of the health systems for diabetes retinopathy screening. Fundus images obtained from the device can be shared among specialists for discussion and identification, hence reduces the need for direct referral of patients to tertiary care units for ‘in person’ examination purposes [7]. In addition, smartphone-based retinal imaging also provides an early exposure for medical students and relevant healthcare trainees on the methods of smartphone-based fundus imaging which is relatively easier than conventional direct fundus imaging [8–10]. Moreover, the operation of smartphone-based ophthalmoscopy can be utilized not only by highly proficient healthcare professionals but also by all healthcare workers [11]. Examples of smartphone-based fundus imaging applications in ophthalmology include diabetic retinopathy screening, optic nerve head evaluation, hypertensive retinopathy evaluation [8], and note progressions of a range of other retinal pathologies, age-related macular degeneration, retinal tumors, and many more [7].

The reason why research on various retinal imaging methods especially ones that are affordable and portable are important is because of the positive impact it has on early diseases prevention. For example, according to Karakaya and Hacisoftaoglu [12], diabetic retinopathy (DR) is the most prevalent cause of blindness or vision loss, resulted by the bleeding of small blood vessels of the retina. As of now, there are 537 million adults worldwide who have diabetes and this is expected to rise to 643 million by 2030 and 783 million by 2045 which is a very alarming rate [13]. The study by [12] further added that current studies have shown that in developed countries, access rate to medical care such as early diagnosis and accurate evaluation of diabetic retinopathy severity ranges from 60 to 90% whereas rates in developing countries are much lower. In other words, patients in low- and middle- income populations are less likely exposed to early detection of diseases that would allow them to get effective treatment in a timely manner.

Over the years, smartphone-based retinal image acquisition devices have shown its credibility in the medical field but only up to a certain extent. Despite being affordable, portable, and able to provide efficient data transfer with expanding internet technology and artificial intelligence in smartphones, professionals still find this method limited to screening purposes only and beneficial only for non-ideal clinical settings [1, 14, 15]. This is because the concept of smartphone-based fundus imaging itself is still incapable of competing against reference standards in terms of image quality and sensitivity. Furthermore, certain existing smartphone-based retinal imaging devices do not come with a universal adapter. For instance, the PanOptic by Welch Allyn, D-Eye by S.R.L., iNview by Volk, and HEINE-iC2 by Heine Optotechnik are only compatible with specific models of smartphone such as the iPhone 5 and iPhone 6 which is a disadvantage since the iPhone 5 and iPhone 6 are no longer iOS supported and are not easily obtained as of now. Among the existing devices in the market, only a few of them are universal such as the Peek Retina by Peek Vision, the adapter Paxos Scope by Digisight Technologies, and the adapter Choroida Fundus Explorer by Choroida. Smartphone-based retinal imaging devices that are only usable with certain types of smartphones limits the purpose of its concept being portable, affordable, and designed for non-ideal clinical settings.

Therefore, this paper aims to develop a universal adapter that is compatible with various smartphone models, especially with currently available smartphone models in the market. Developing this universal adapter would broaden the usability of existing smartphone-based retinal image acquisition devices as it then would allow users operate these devices directly using the smartphone models that they have. A detailed experimentation is needed to be designed to ensure the universal adapter does not compromise the quality of images taken.

### Smartphone-based retinal imaging system

Smartphone-based retinal imaging system involves high-quality cameras and high-resolution video features from the smartphone itself that can be utilized to achieve portable and affordable retinal imaging [7]. The role of smartphones for retinal imaging is becoming more significant with the deep integration of internet technology, expanding camera quality, and implementation of artificial intelligence in smartphones. In addition, smartphones-based retinal image acquisition devices have a prominent potential in becoming the first choice for direct ophthalmoscopy in non-ideal clinical settings for instance in geographically remote areas, or even emergency rooms [16, 17]. A study by Debuc [18] had stated the utility of smartphone-based retinal image acquisition devices has shown a steady increment with the recent advancements not only on the speed and coverage of wireless networks specifically in rural areas, but also on the power consumption efficiency of these devices that allows longer battery life. It was also stated in the study that in medical practice, 80% of doctors now utilize smartphones and mobile devices [18]. Another study had also estimated that one out of two physicians incorporates either a smartphone or a personal digital assistant (PDA) in their work and this pattern is expected to increase [16]. However, these devices are yet to be fully applicable as quality diagnostic tools, hence they are limited to screening purposes only as of now [15].

**Fig. 1:**
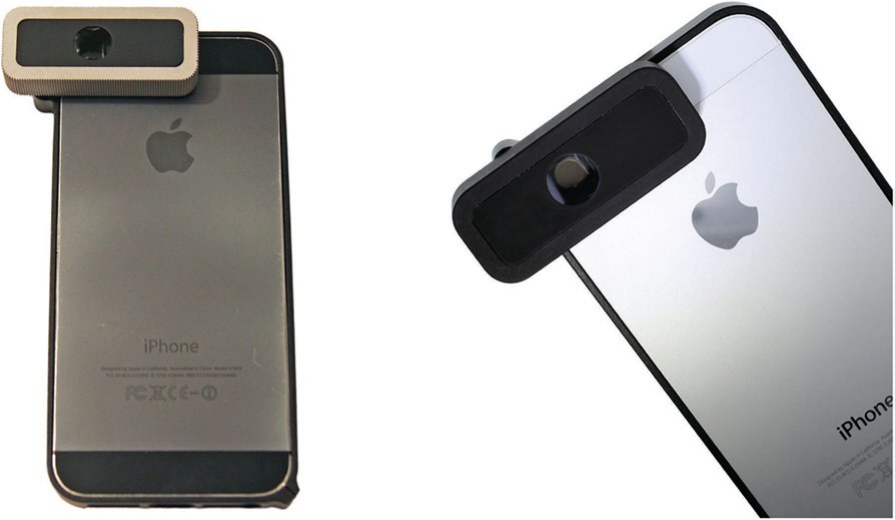
D-Eye prototype attached to an iPhone 5 and iPhone 5 s from the left [19]

Based on research conducted, smartphone-based retinal image acquisition devices are available in two forms, which are a full device or an adapter. Several smartphone-based retinal image acquisition devices are currently available in the market such as the D-Eye, PanOptic, iNview, Peek Retina, HEINE-iC2, Paxos Scope, and Choroida Fundus Explorer. The D-Eye, PanOptic, iNview, Peek Retina, and HEINE-iC2 are full devices meanwhile the Paxos Scope and Choroida Fundus Explorer are adapters. Figure 1 shows the D-Eye attached to an iPhone that was developed by Russo et al. [19].

**Fig. 2:**
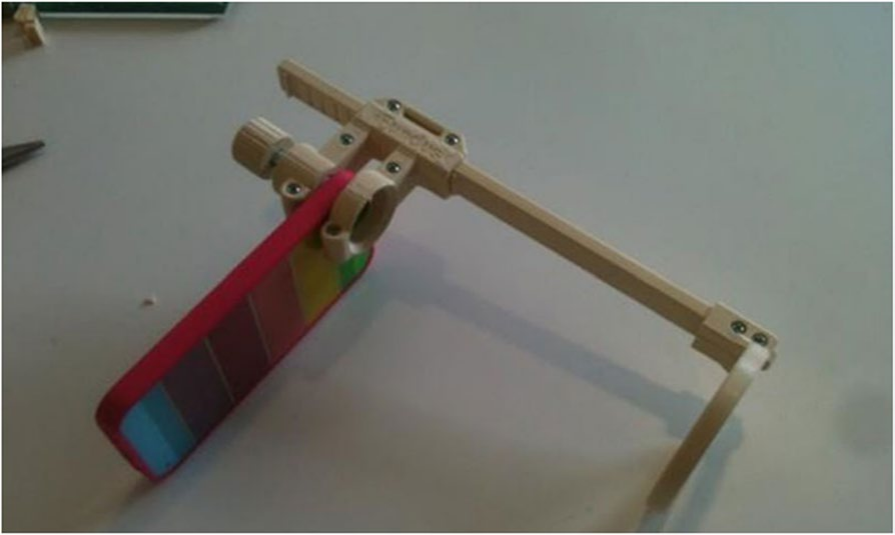
3D-printed retinal image acquisition adapter [20]

Adapters are considered to be simpler to operate as it does not have an internal optical configuration, unlike full devices. Furthermore, adapters are more affordable as most of them are 3D-printed that are combined with off-shelf components such as bolts and nuts [20–22]. Adapters typically comprise of two holders connected, one is the phone holder, and one is the lens holder. Figure 2 shows a 3D-printed adapter developed by Hong et al. [20]. The adapter was designed using a software named as SolidWorks Professional which is a computer-aided design (CAD) software. Besides this, the adapter is universal which means it can be used with different types of smartphones. A typical retinal imaging in smartphone utilizes a 20 dioptre (20D) condensing lens which applies the same concept as indirect ophthalmoscopy. In most cases, illumination source is required for pupil dilation to acquire the retinal images. The 20D condensing lens are the component responsible for focusing the fundus while an image of the focused fundus starts to appear on the smartphone display screen. For efficient acquisition of retinal images, sufficient training is required to be able to obtain the optimum filming distance to the point at which the fundus is focused [7]. Once the desired fundus image is seen from the display screen, the image of the focused fundus is captured, and laterally inverted and upside down. Moving the smartphone closer to the patient allows for a magnified glimpse of a retinal lesion. This is paired with the 20D lens’ relative movement away from the patient. Similarly, the smartphone is positioned at a further distance from the patient in order to have a larger field of view. Different quadrants of the retina can be observed in the same way as in indirect ophthalmoscopy by shifting the patient’s gaze [7]. Stated in the study by Iqbal [7], there are two main factors coming from the smartphone that could affect the efficiency of fundus focusing which are the position of camera lens of the smartphone and the location of illumination source.

### Smartphone compatibility comparison of existing smartphone-based retinal image acquisition devices/adapter

Researchers and companies involved in clinical equipment manufacture have proposed and developed portable, easy-to-use, and affordable smartphone-based retinal image acquisition device/adapter with the objective of obtaining high-quality retinal images. This section discusses the smartphone compatibility of existing smartphone-based retinal image acquisition devices or adapters that are both commercialized and non-commercial. Table 1 shows a summary of existing commercial and non-commercial smartphone-based retinal image acquisition adapters and devices, along with its type and smartphone compatibility. For research purposes, an unnamed device developed by Myung et al. [22] that is listed has been labelled as Alpha.

**Table 1:** Summary of smartphone compatibility comparison of existing smartphone-based retinal image acquisition devices/adapter

Commercial Group	Device Name	Specifications	
		Type	Smartphone Compatibility
Commercial	Peek Retina by Peek Vision	Full device	Universal
	PanOptic by Welch Allyn	Full device	iPhone 6/6 s/6 plus only
	D-Eye by S.r.l	Full device	iPhone 5/5 s/6/6 s/6 plus/6 s plus/SE/7 only
	iNview by Volk	Full device	iPhone 5/6/6 s only
	HEINE-iC2 by Heine Optotechnik	Full device	iPhone 5/5 s/SE/6/6 s/7/8 only
	Paxos Scope by Digisight Technologies	Adapter	Universal
Non-commercial	Choroida Fundus Explorer by Choroida	Adapter	Universal
	CellScope Retina by Kim et al., [23]	Full device	iPhone 5 s only
	Alpha by Myung et al. [22]	Adapter	iPhone 5 only

From the comparison, it is found that for most existing devices, selection of compatible smartphones is very limited which is a significant disadvantage. For example, the iNview are only compatible with the iPhone 5 and iPhone 6 series, meanwhile the PanOptic is only compatible with the iPhone 6 series, and the D-Eye is only compatible with the iPhone 5, iPhone 6, and iPhone 7. Among the listed commercial devices, the Paxos Scope, Peek Retina, and Choroida Fundus Explorer are universal. Both the Peek Retina and the Choroida Fundus Explorer use the concept of ‘clip-on’ that allows it to be easily attached onto smartphones. This concept provides the flexibility in positioning the smartphone for camera alignment. Similarly, the Paxos Scope is also universal as the device’s smartphone holder are adjustable to fit the variety sizes of smartphones. For the non-commercial devices, both devices were developed with the idea of being universally compatible as one of the objectives. However, in respective studies, both studies tested their devices using one type of phone model only. An iPhone 5 s was used with the CellScope Retina [23], and an iPhone 5 with Alpha [22]. These smartphone models are no longer in production and supported by the latest iOS due to the internal components of the models are unable to handle the iOS effectively. In addition, the camera quality of these models is low, considering the high-quality cameras that the latest smartphones models have. The camera system for latest smartphones provides broader functionality for instance more advanced lighting and magnification control. In addition, latest smartphones can be acquired easier compared to models that are no longer being produced by manufacturers. Furthermore, it is also observed that most of the listed smartphone-based retinal image devices listed in Table 1 are only used or tested with iPhones where no android smartphones were involved which further shows the limitation of existing devices. This may be due to iPhones are more popular among healthcare workers. Several studies on the usage of smartphone in clinical practices such as [24] and [25] have found that there are more iPhone users over other smartphones in clinical practice.

As conclusion, most existing devices have restricted usability. For instance, the PanOptic, D-Eye, iNview, and HEINE-iC2, are only compatible with Apple’s smartphones, which are also from older generations for instance the iPhone 5, 6, 7, and 8. These models are no longer in production and the current iOS operating system is also not supported on these models. This suggests that future smartphone-based retinal image acquisition devices should be made universal, where the adapter to hold the smartphones is compatible to most smartphone models which helps to broaden its usability.

**Fig. 3:**
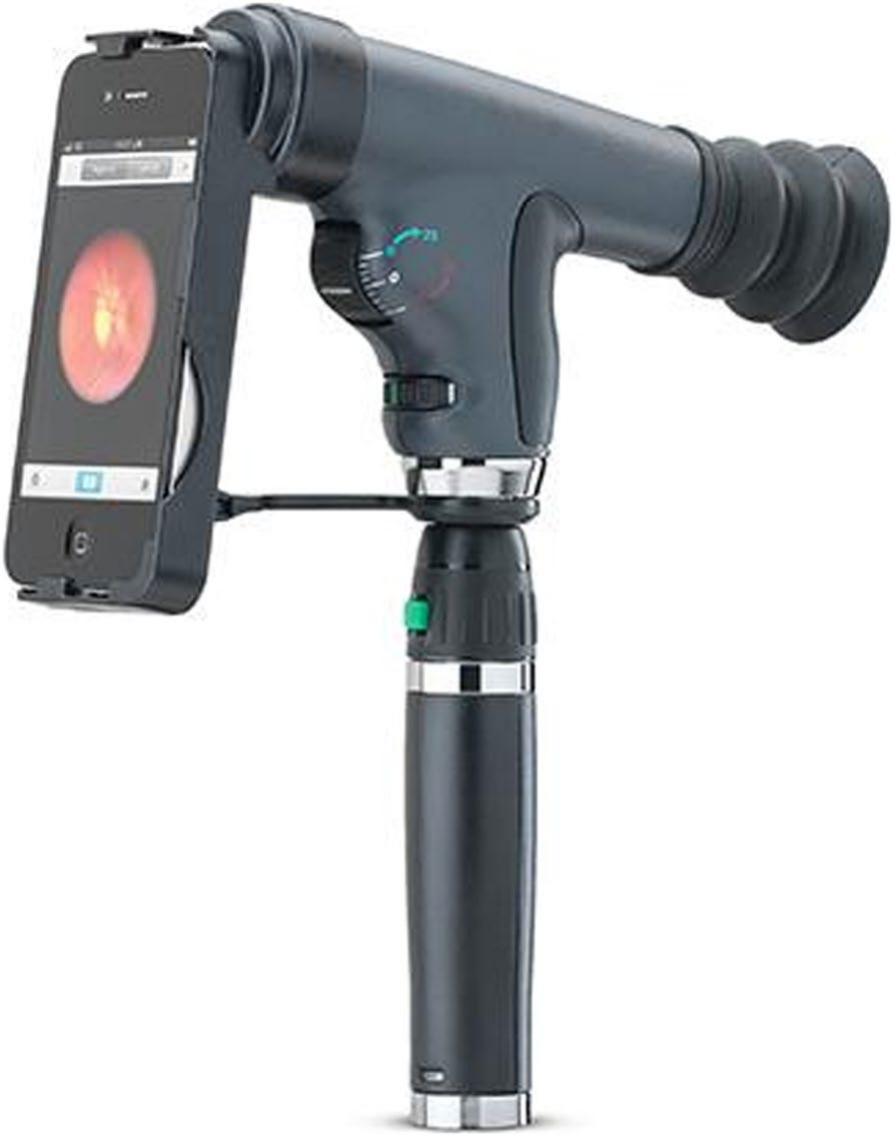
Assembly of PanOptic with its original adapter [32]

**Fig. 4:**
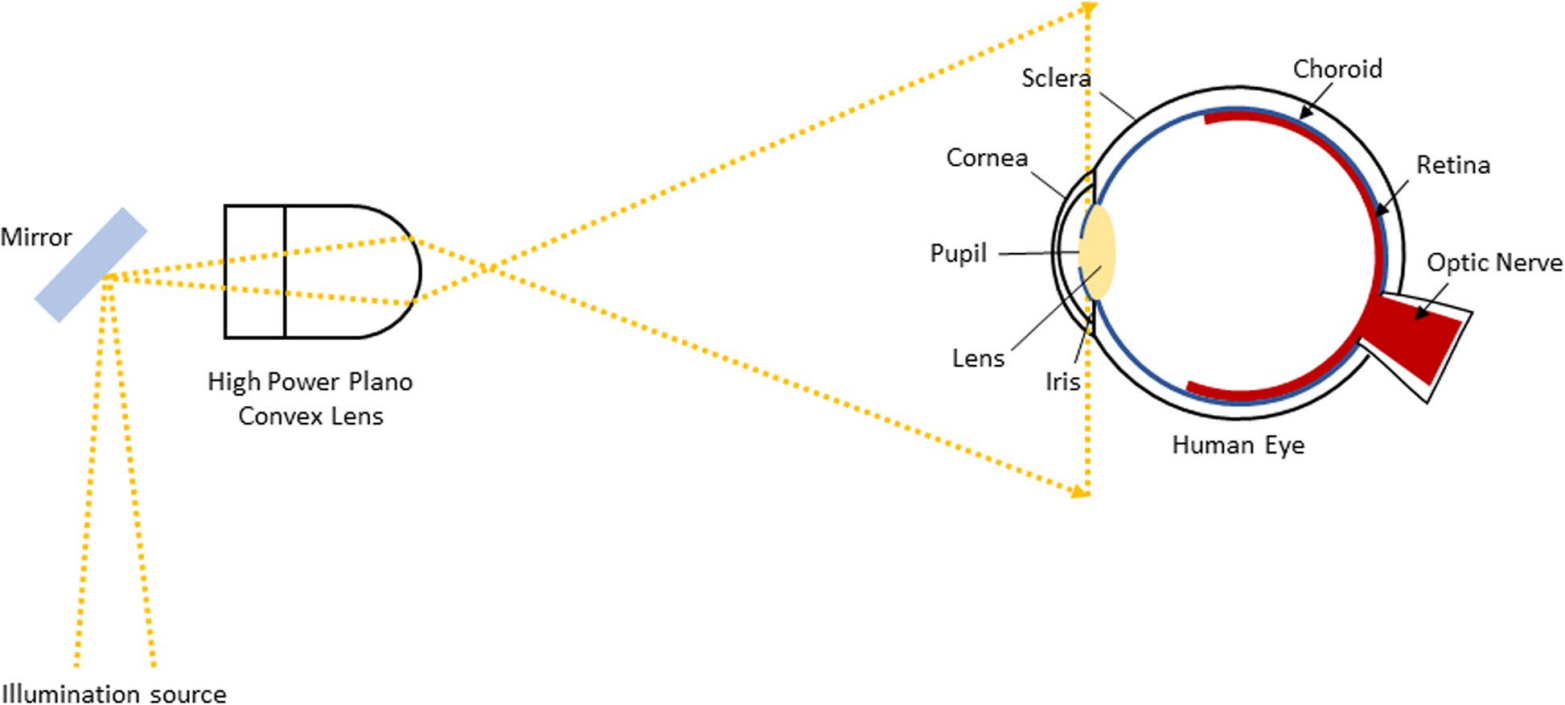
Redrawn schematic ray diagram of PanOptic Ophthalmoscope adapted from [31]

Current studies shown on the PanOptic ophthalmoscope developed by Welch Alyn have obtained different findings on its efficiency [26–31]. Figure 3 shows the assembly of PanOptic with its original adapter [32]. The PanOptic provides approximately a field of view of 25° which is considered small due to its optical configuration. Figure 4 demonstrates a redrawn ray diagram of the PanOptic ophthalmoscope adapted from [31]. The field of view is small because the high-power Plano convex lens used in the device diverges the illumination source widely hence only a small amount of light can enter the pupil resulting in a poor red reflex [31]. The developer of the PanOptic ophthalmoscope has stated that the device allows retinal imaging without dilation. However, a study conducted by [26] have found that field of view provided by the ophthalmoscope was not large enough, where even the optic disc and macula was not seen in any of the images taken and suggested further testing to determine the best way to utilize the PanOptic ophthalmoscope optimally. The study by [12] have also found that the PanOptic ophthalmoscope has the lowest classification accuracy of 61% when compared to other existing commercial smartphone-based retinal image acquisition devices such as the iNview, Peek Retina, and D-Eye.

However, the study by Mccomiskie et al. [28] involving eight medical students have provided statistical analysis that shows the PanOptic ophthalmoscope is preferred over the conventional ophthalmoscope for the ‘ease-of-use’ despite the quality of image acquired were much poorer. The study by Hu et al. [27] have used the PanOptic ophthalmoscope together with a slip-lamp translator and a rest system for patient’s chin and head which greatly improves stability but caused increment in the cost overall. In addition, Hu et al. [27] further stated that the software in the iExaminer application caused the resolution of fundus images acquired is low. The iExaminer application also captures images by selecting stills from video recorded which also lowers the quality image taken. This suggests the possibility of better image quality taken if the image is taken directly and using the smartphone’s default camera application. Furthermore, the study by Petrushkin et al. [30] have also compared the performance of PanOptic ophthalmoscope and a direct ophthalmoscope and have found that the PanOptic ophthalmoscope has higher sensitivity and specificity. Hence, the PanOptic ophthalmoscope still shows its potential as a portable and low-cost smartphone-based retinal image acquisition device which suggest that there is still room for improvement.

## Methods

**Fig. 5:**
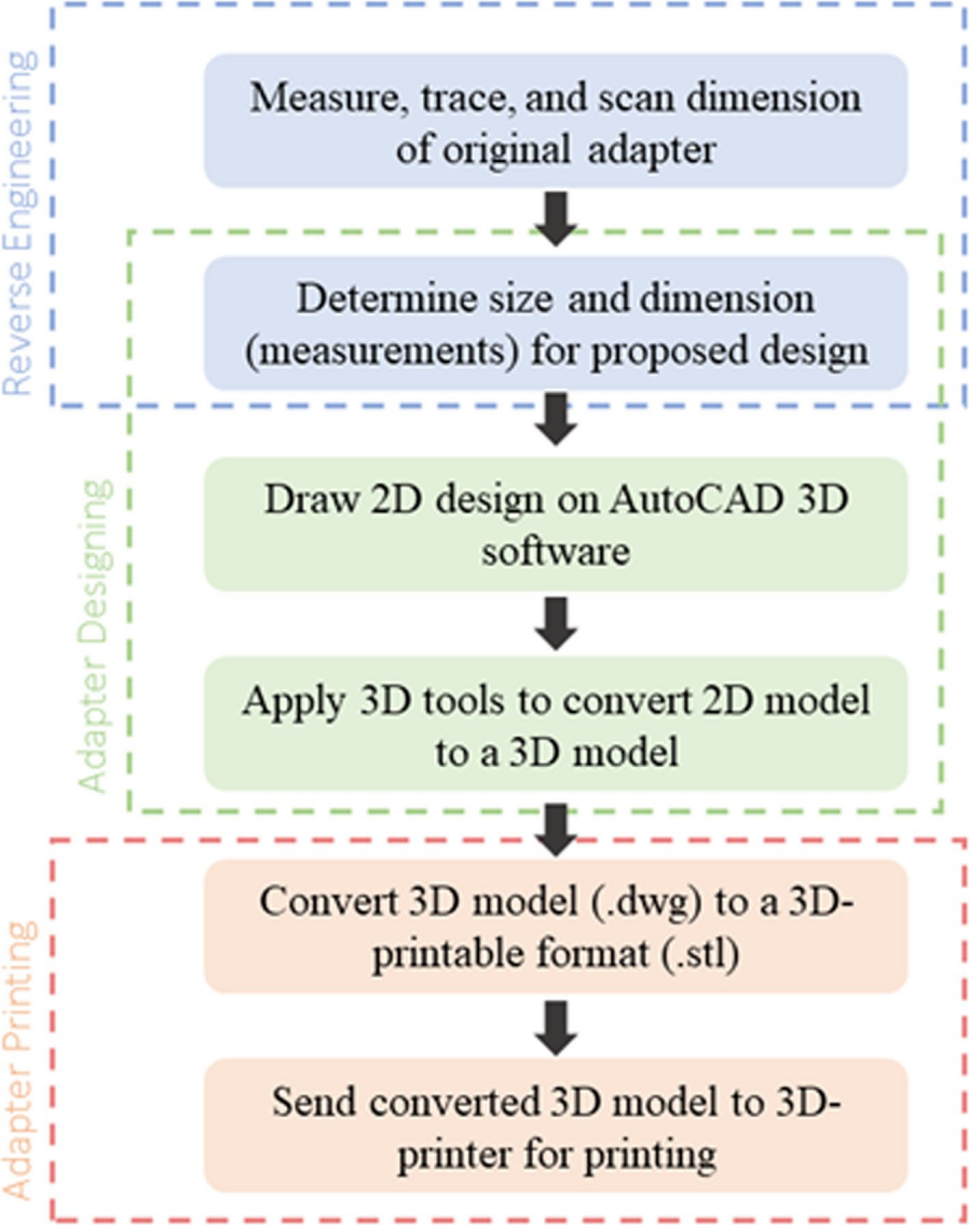
Process of adapter development

A personal computer with the specifications of Intel(R) Core™ i5 12400F processor for CPU, 16 GB for Random Access Memory (RAM) and 64-bit operating system powered by Windows 10 were utilized. Besides this, three software were selected to perform this project which are AutoCAD 3D, Autodesk Inventor, and Ultimaker Cura. Furthermore, a few tools and materials were used for this project. For 3D-printing, Ender 3 V2 3D-printer by Creality was utilized along with polylactic acid (PLA) as the filament. For measurement purposes, a pair of Vernier Caliper was used. For this project, the PanOptic Ophthalmoscope was selected to be experimented with and improved based on the research conducted. The development of the adapter consisted of three main processes which started with reverse engineering, followed by adapter designing, and lastly adapter printing. Figure 5 shows a flowchart that summarizes the process of adapter development. Each step is further discussed in respective sections.

Table 2 summarizes the optical and mechanical parameters of the PanOptic Ophthalmoscope according to its manufacturer and Karakaya and Hacisoftaoglu [12]. The development procedure and experimental design proposed were constructed with the objective to determine the measures that could be done to optimally utilize the PanOptic ophthalmoscope and improve mainly on the smartphone compatibility, and type of data collected while maintaining its primary features such as its practically, portability, and low-cost.

**Table 2:** Specification of PanOptic ophthalmoscope

Properties	Description
Field of view	25°
Dilation dependency	Not required
Smartphone compatibility	iPhone 6/6 s/6 plus only
Application	Has own application named as iExaminer
Type of data collected	Image stills from recorded video
Cost	Considered to be much affordable when compared with conventional retinal imaging devices
Other features	- Has a focusing wheel to adjust focus level - Has an aperture/filter dial to control aperture level or type of filter

### Reverse engineering and adapter designing

The main criterion of the proposed adapter is to have a universal smartphone compatibility. As stated previously, the original adapter provided by the manufacturer of the PanOptic ophthalmoscope could only be used with the iPhone 6 series. The iPhone 6 series has been long discontinued and can no longer be obtained. Without a universal smartphone adapter, it would not be possible to use the PanOptic. Hence, the first step in developing the universal adapter is reverse engineering to understand the mechanism of the original adapter and identify the required changes.

**Fig. 6:**
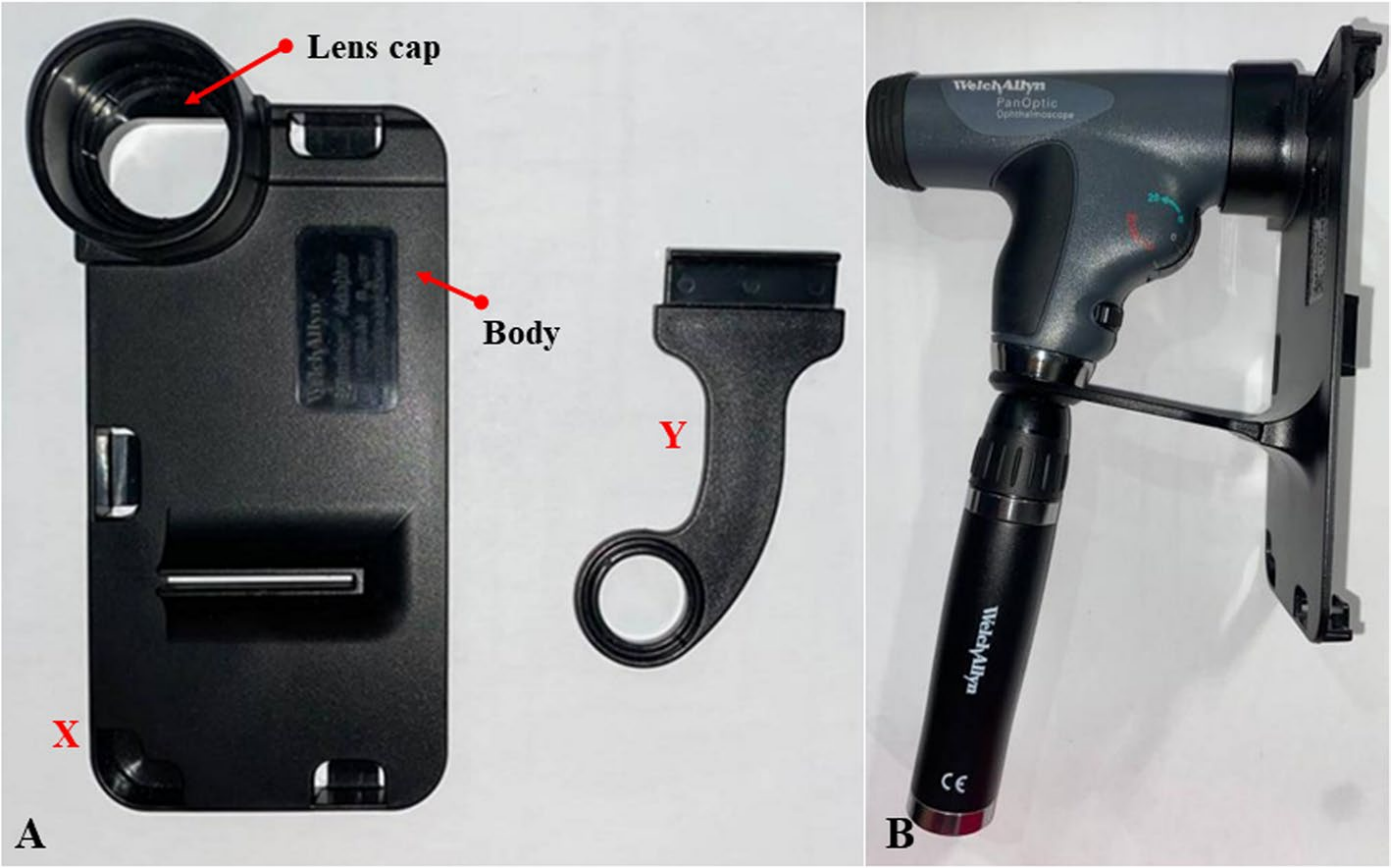
Original adapter of PanOptic ophthalmoscope. **a** Main phone holder labelled as X and connecter labelled as Y. **b** Assembly of the adapter with the ophthalmoscope

**Fig. 7:**
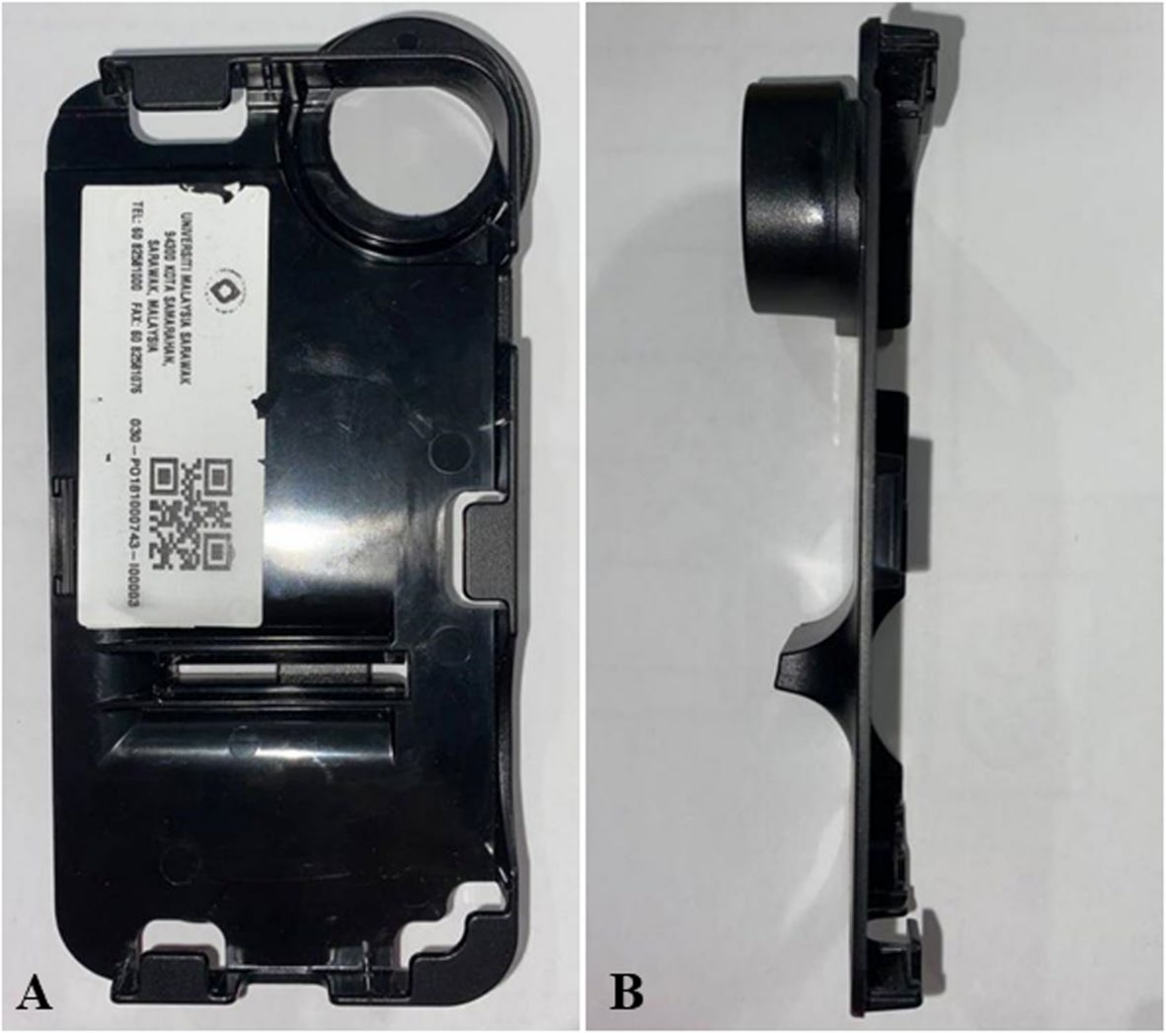
Different viewing of the original adapter. **a** Front. **b** Side

For this purpose, the original adapter was divided into two main parts, which are the lens cap and the body as shown in Figs. 6 and 7. Referring to Fig. 6, the original adapter provided by the developer of the PanOptic ophthalmoscope consists of two parts, which is the main phone holder labelled as X and the connector labelled as Y. The connector provides extra support and grip between the smartphone and the device. The main holder comprises of the lens cap and the main body. Figure 6b shows how the adapter is assembled onto the ophthalmoscope. Figure 7 shows the front view and side view of the adapter that shows that the phone holder has a fixed dimension for an iPhone 6 which is 131.10 mm in height, 67.0 mm in width, and 6.90 mm in depth. Besides this, the distance between the surface of the eye piece lens and camera opening of the original adapter was measured to be approximately 12.4 mm.

**Fig. 8:**
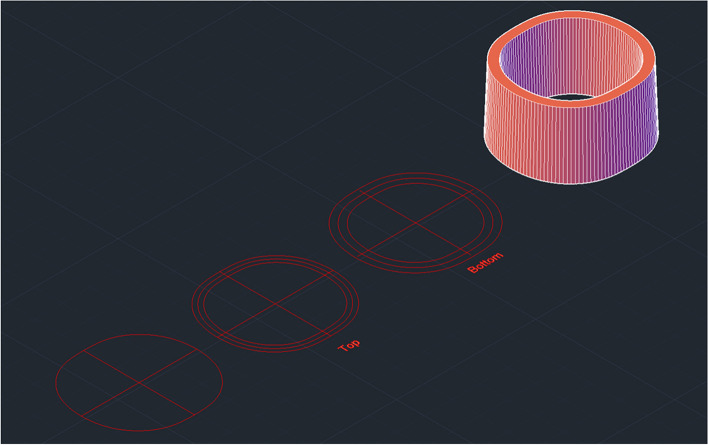
Visualization of lens cap traced, scanned, and developed into a CAD model

**Fig. 9:**
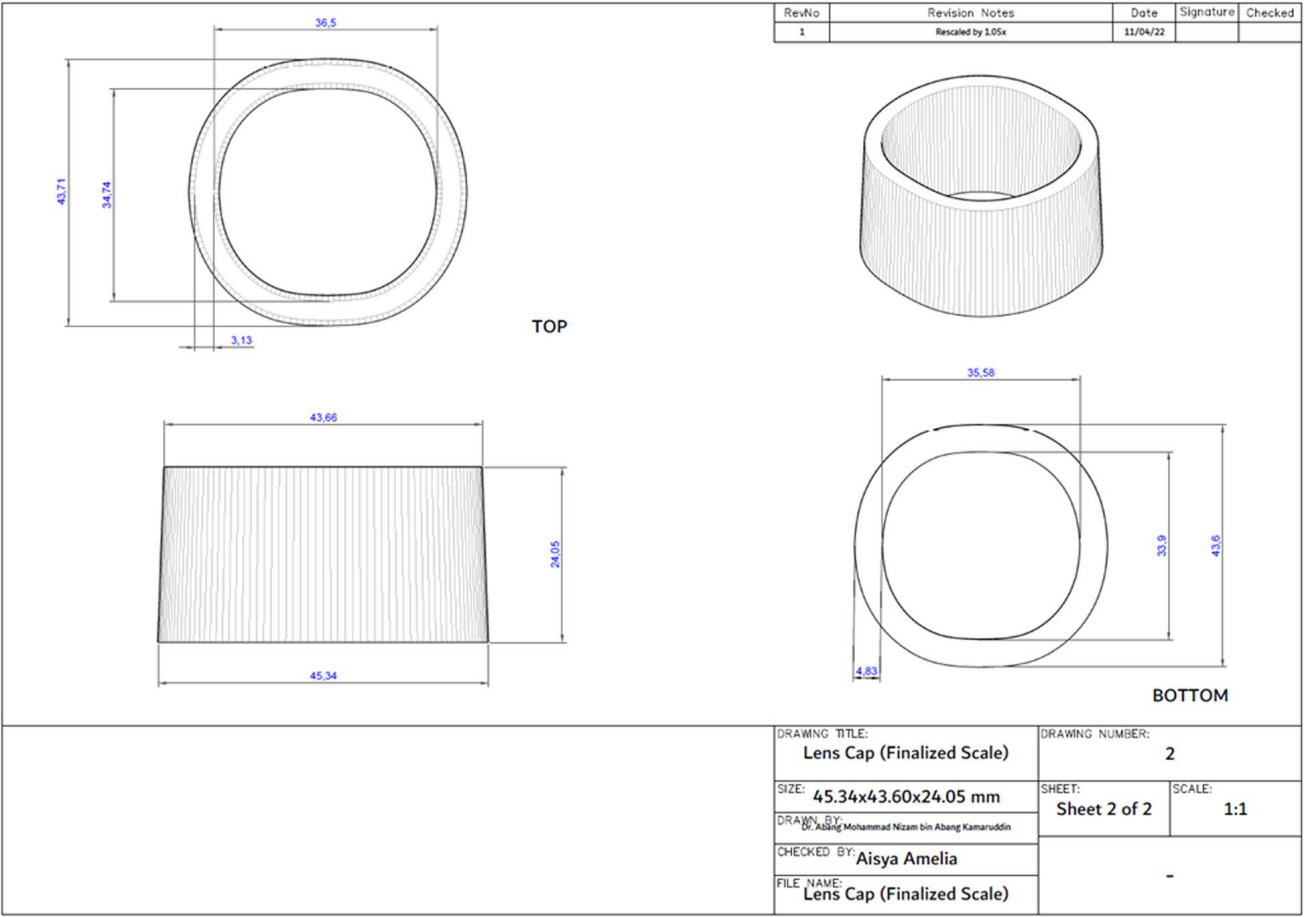
Technical drawing of finalized lens cap

Upon measuring the original adapter, the lens cap was identified to have a specific shape and have different thickness in certain parts of the lens cap. Hence, tracing was employed. The measurement obtained from tracing was then scanned into the AutoCAD 3D software to generate a CAD model. Once the CAD model of the lens cap was obtained in 2D, it was converted into a 3D model using the 3D tools available in AutoCAD 3D. The completed 3D model which was in*.dwg* format was then converted to a printable format known as*.stl* for the 3D-printer to recognize. This conversion process is known as ‘slicing’. The process of drawing the 2D and 3D models, and format conversion was repeated until the desired dimension of lens cap was achieved to proceed with proposing designs for the body of the adapter. Figure 8 shows a representation of the traced lens cap dimension which was developed into a CAD model meanwhile Fig. 9 shows the technical drawing of the finalized lens cap. The completed 3D-model of the lens cap was printed to test with the PanOptic which involved several adjustments and printed prototypes. Only once the right dimension of the lens cap was obtained, the main body of the adapter was designed. The finalized lens cap has different thickness and diameter at the top and bottom. The top has a thickness of 3.13 mm meanwhile the bottom has a thickness of 4.83 mm. The top vertical and horizontal inner diameter is 36.50 mm and 34.74 mm respectively meanwhile the bottom vertical and horizontal inner diameter is 35.58 mm and 33.90 mm.

Designing the body of the adapter involved the same steps except the tracing and measuring using the Vernier callipers. The size and dimension of the adapter’s body was measured with reference to the average dimension of a smartphone. The designing process of the body had resulted in four prototypes produced where prototype testing was conducted to check the stability and usability of each prototype when assembled with the PanOptic and a smartphone. For this purpose, several smartphones were tested which were an iPhone 11, Huawei Y9, iPhone 14, and Google Pixel 6. All four smartphones have different dimensions and camera positions. For instance, the Huawei Y9 has only one lens which is located at the top left of the smartphone, and the smartphone has a dimension of 162.40 mm, 77.10 mm, and 8.05 mm (height × width × depth). Both the iPhone 11 and iPhone 14 have two camera lenses (wide and ultra-wide) which are located at the top left. However, the camera lenses of the iPhone 11 are in vertical order meanwhile the camera lenses of the iPhone 14 are placed diagonally to one another. The dimension of the iPhone 11 and iPhone 14 are 150.90 mm × 75.70 mm × 8.30 mm and 146.70 mm × 71.50 mm × 7.80 mm respectively. It is also noted that the camera lenses of the iPhone 14 are thicker (taller bumps) when compared to the iPhone 11. Lastly, the Google Pixel 6 also has two camera lenses (wide and ultra-wide) positioned at the top left, but the ultra-wide camera lens is closer to the center of the smartphone. Furthermore, the camera lenses of the Google Pixel 6 have the tallest bump and are designed in a horizontal beam. The Google Pixel 6 has a dimension of 158.60 mm × 74.80 mm × 8.90 mm. Once the developed adapter is determined to be compatible with the PanOptic, further performance evaluation was conducted.

### Process of 3D-printing

The method for 3D-printing used in this project is fused deposition modeling (FDM) which is a technology involving melt extrusion for the deposition of filaments made of thermal plastics to form assigned pattern [33]. According to a study by Kumar et al. [34], the FDM method distributes the 3D-printing into two materials. The first material is to build the main part of the 3D model meanwhile the second material is to produce a disposable structure that acts as support for the 3D model. The layer thickness is set to 0.20 mm which is an ideal height for optimized printing [35]. Table 3 summarizes the properties used for 3D-printing in this project. To start 3D-printing, the printing bed and filament are first heated for a few minutes before begin printing. Secondly, the printing bed is levelled optimally. To level the printing bed, simply use a piece of paper, place it under the printing nozzle, and gently move the paper underneath and feel the grip. The grip should not be too tight, or too loose. If the grip on the paper appears to be tight, the printing bed should be lowered. Vice versa, if the paper appears to be loose, the printing bed should be raised. Once this process is completed, the 3D-printing can begin.

**Table 3:** 3D-printing settings of Creality Ender 3 V2

Process	Fused Deposition Modeling (FDM)
Nozzle Size (mm)	0.4
Layer Thickness (mm)	0.2
Infill (%)	20
Material	Polylactic acid (PLA)
Filament Size (mm)	1.75

### Proposed performance evaluation

The first performance evaluation conducted was a stress test which was performed using Autodesk Inventor software to analyse the physical stability of the adapter. The method used for the second evaluation which is for statistical analysis was Subjective Image Quality Assessment involving a non-mydriatic clinical testing. The assessment on images collected was evaluated using the 5-point scale which is based on the FOTO-ED studies [26, 36, 37]. In addition, an end-user survey was prepared to collect feedback of the device in terms of compatibility, stability, usability and repeatability, and suitability of the device in real clinical settings.

**Fig. 10:**
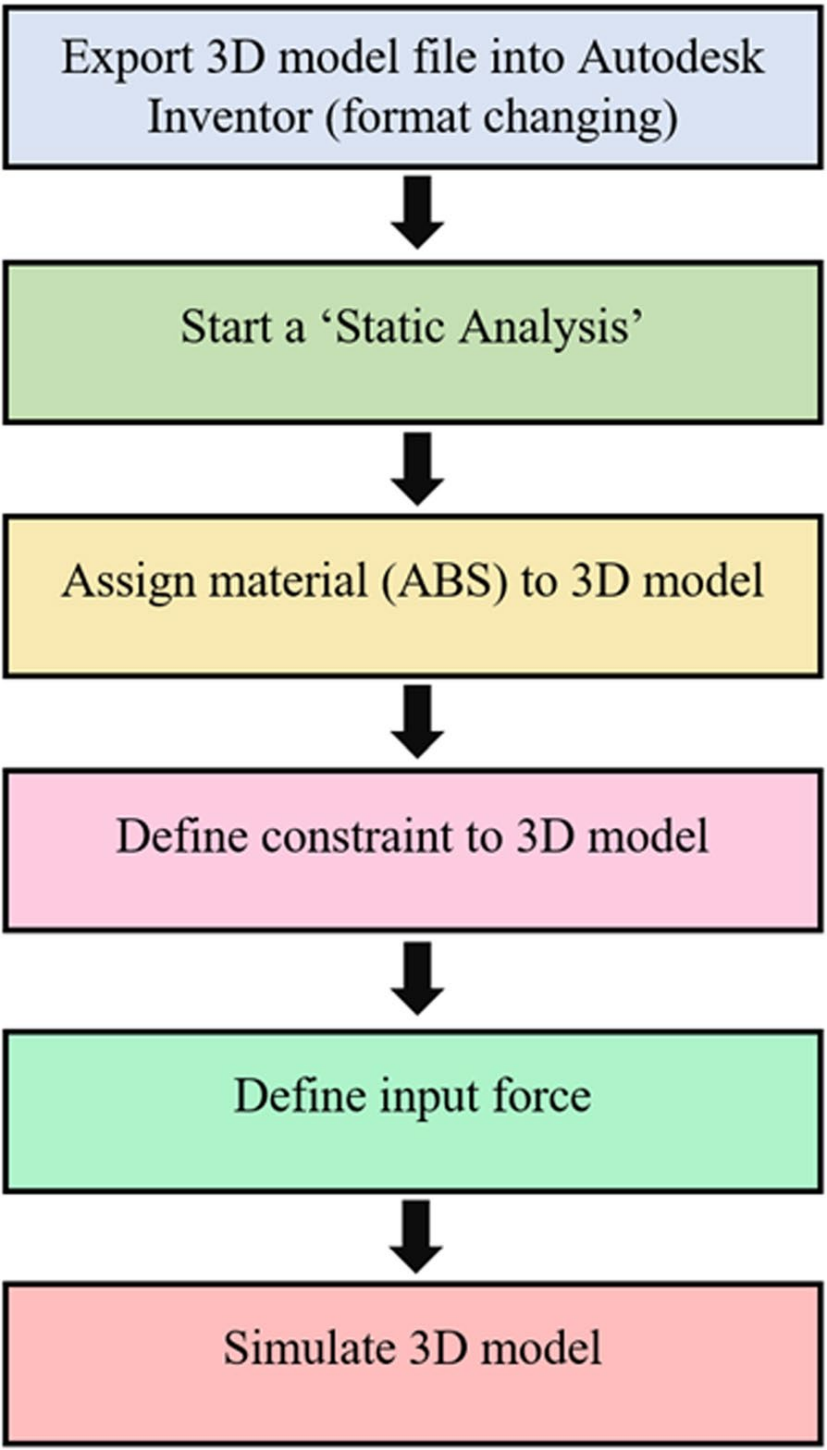
Process of stress simulation using Autodesk Inventor

For the stress analysis, the finite element analysis (FEA) method was applied using the Autodesk Inventor software. Finite element analysis is known as a numerical method used to calculate strains and stress in describing the behaviour of elastic solids [38]. The main purpose of performing stress analysis was to observe and evaluate the possible forms of deformation of proposed adapter in terms of stress, displacement, and strain. This helps to identify which areas are subject to excessive stress when force is applied. Each part was evaluated individually but focus was on the main body and the movable handle as the handle cover only functions as a cover for the movable handle. Figure 10 shows the flowchart that summarizes the process of stress simulation.

To begin the simulation, the 3D model file was first exported into the Autodesk Inventor which converts the*.dwg* format to a format that allows the software to recognize the 3D model. The export process involved several steps in ensuring the CAD model was converted correctly into the Autodesk Inventor. Next, a static analysis was selected where the main input force was not affected by time, temperature, or atmosphere pressure. The next step was assigning a material to the 3D model. The filament used to print the adapter was polylactic acid (PLA). PLA is a type of biodegradable thermoplastic that is derived from renewable resources and has mechanical properties suitable for 3D-printing such as high strength and high stiffness [39]. In the materials library of the software, PLA was not listed, thus, a new material was created where the mechanical and thermal properties of the PLA was set with reference to the average values of PLA provided in MatWeb which is an online service that provides database of engineering materials such as thermoplastic, metals, ceramics and more based on existing products in the market where data and specification sheets are supplied by manufacturers and distributors. Table 4 summarize the properties of the PLA material.

**Table 4:** Properties of PLA material

Name		PLA
General	Mass Density	1.280 g/cm3
	Yield Strength	40.100 MPa/ 5.816 ksi
	Ultimate Tensile Strength	62.900 MPa/ 9.122 ksi
Stress	Young’s Modulus	2.270 GPa
	Poisson’s Ratio	0.3

**Fig. 11:**
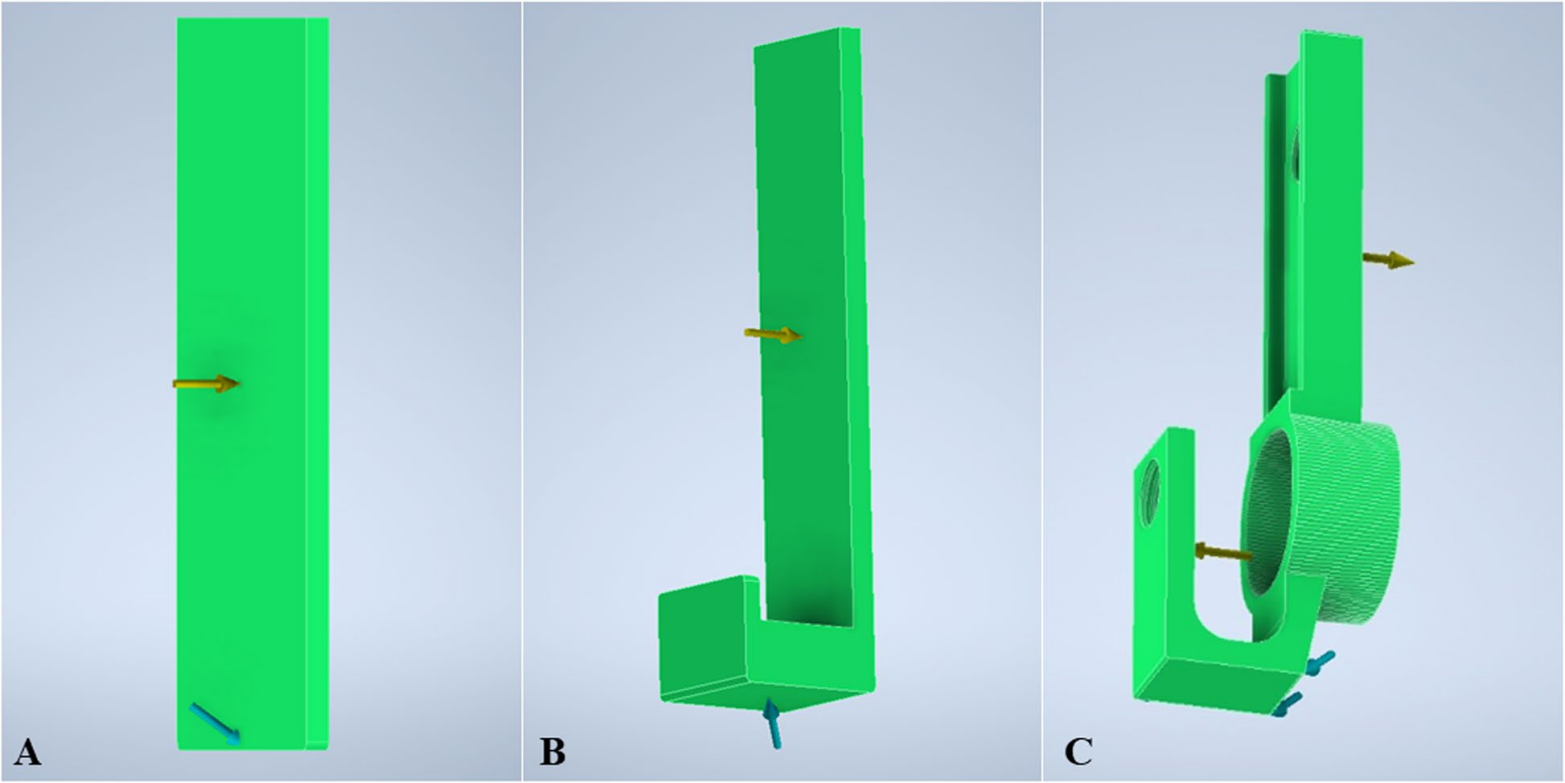
Location of constraint displaced. **a** Handle cover. **b** Movable handle. **c** Main body

Next step was defining the constraints of the 3D model by identifying the location of the adapter that will be restrained from any movement, displacement, or rotations [38]. This was to establish relationships between components in an assembly that helps to control its position and behaviour. As an example, Fig. 11 shows the location of constraint for the handle cover, movable handle, and main body. Arrow in blue represents the constraint placement meanwhile arrow in yellow represents the load placement. The following step was defining the external force that is going to be exerted on the adapter. In this experiment, a singular force of 5 lbf was applied. After that, the stress analysis was simulated. In this experimentation, the stress evaluation on proposed adapter was conducted based on von Mises stress and 1st principal stress. Von Mises stress is also known as equivalent stress. The criterion of von Mises states that if the von Mises stress of a material under a load is equal or greater than its yield limit under a simple tension, then the material will yield [40]. As for the 1st principal stress, it is to observe the areas experiencing the maximum tensile stress induced on the adapter under applied load.

For the second performance evaluation, the Subjective Image Quality Assessment was conducted through retinal imaging using the developed adapter together with the PanOptic ophthalmoscope. The non-mydriatic clinical testing was conducted to collect retinal images from real patients. A total of 5 experts (3 ophthalmologists and 2 optometrists were selected as examiners for the clinical testing. The PanOptic ophthalmoscope together with the developed universal adapter and Google Pixel 6 smartphone were utilized. A total of 14 patients between the ages of 20 and 80 were recruited as subjects. Full written informed consent was obtained from both the examiners and patients. A total of 28 eyes were imaged (left and right) and recorded in video format where the best images from each eye were selected (by frame) by the examiners for review and grading. For the review and grading of images, six experts were selected which includes the same experts from the clinical testing, one postgraduate medical student, and one associate professor doctor from the Medicine and Health Sciences Department. The method applied to review the images collected was the method used in the study by Day et al. [26] where the 5-point scale was used to grade the quality of images taken [26]. Table 5 shows the 5-point scale with its description where Grade 3 and above is considered as clinically adequate (CA) [28, 31].

**Table 5:** 5-point grading level with its description [26]

Scale	Grade Description
Grade 1	Fundus is not visualized at all
Grade 2	Fundus visualized but the optic disc is not clear
Grade 3	Optic disc is clearly visualized
Grade 4	Optic disc and its surrounding structures are clearly visualized
Grade 5	Clear view of both the optic disc and macula

Besides this, the end-user survey was structured using a Likert scale (1 = poor, 2 = fair, 3 = good, 4 = very good, 5 = excellent). A Likert scale is defined as a type of psychological measurement tool comprising various options for respondents to express their opinions or viewpoints regarding a specific issue or topic in a scientifically accepted and validated method [41, 42]. The frequency scores on the compatibility, stability, usability and repeatability, and suitability of the device were calculated using Microsoft Excel based on the scores obtained from the end-user survey. For the retinal imaging process, the eye rubber cup that was provided together with the PanOptic ophthalmoscope is also utilized according to the manual.

## Results

### Adapter development

**Fig. 12:**
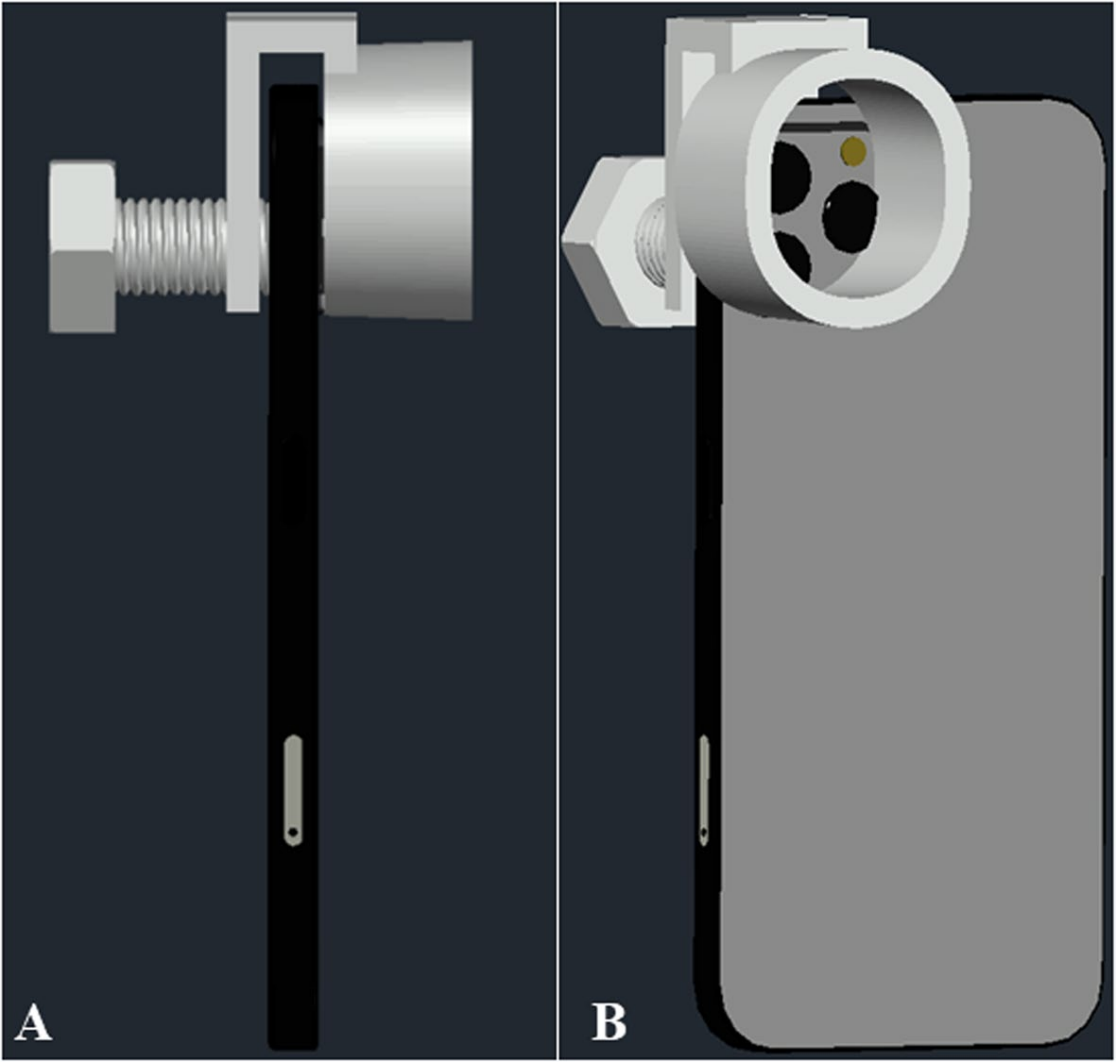
Concept of first prototype designed in AutoCAD 3D. **a** Sideview. **b** Angled perspective

**Fig. 13:**
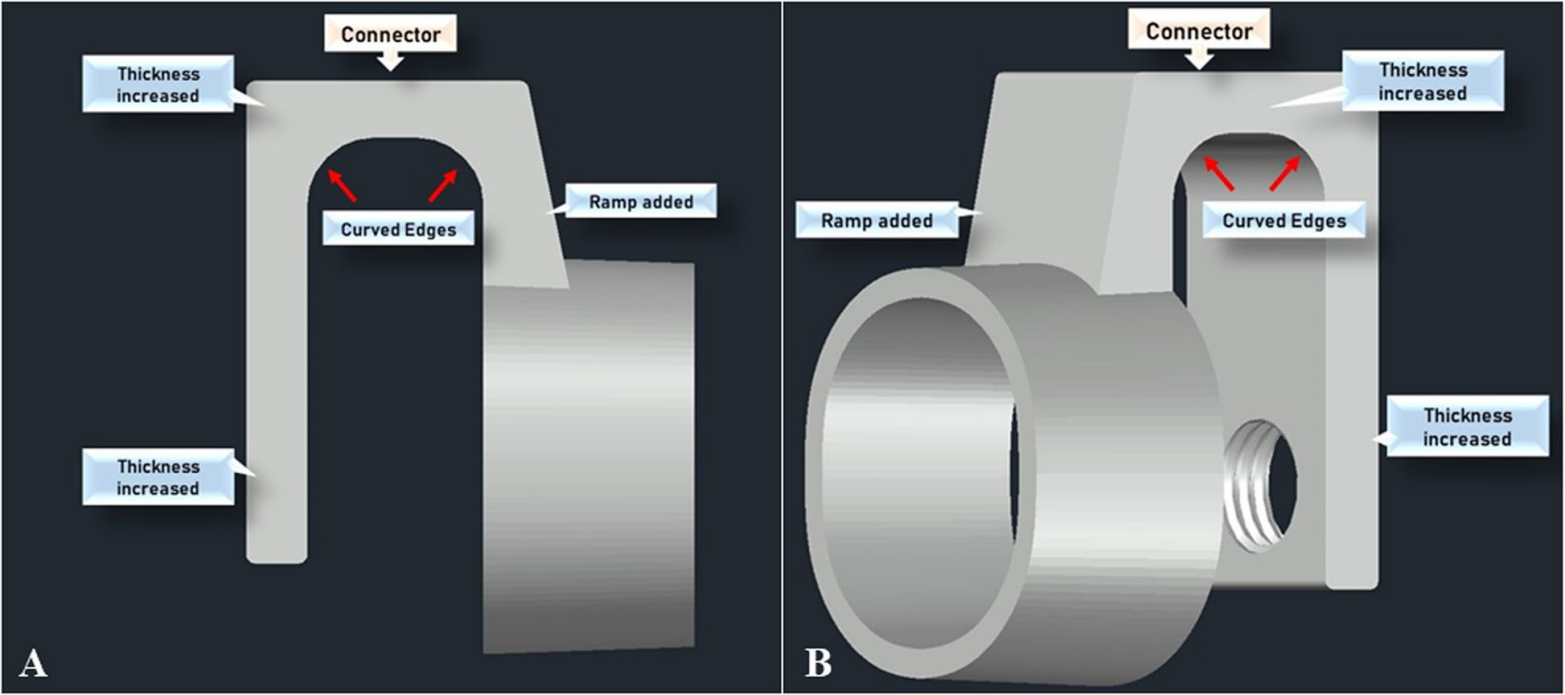
Concept of second prototype designed in AutoCAD 3D. **a** Sideview. **b** Angled perspective

Figures 12 and 13 shows the concept design of the first and second prototypes prepared using AutoCAD 3D. All three prototypes apply a screw system to secure the alignment of the smartphone’s camera onto the PanOptic. The iPhone model used as shown in Fig. 12 is obtained from GrabCAD for visual purposes only. An additional image file shows the dimension of the screw [see Additional file 1]. Referring to Fig. 12, the first prototype has a simple design structure that uses a screw system to secure smartphones onto the adapter and the PanOptic ophthalmoscope. During the testing stage, the adapter got easily broken and thus was analysed. From the analysis, it was identified that the main cause of the defect was the adapter thickness being too thin. It did not have enough surface area in contact with one another to hold the adapter together hence it is fragile and gets easily broken. Furthermore, due to the nature of how 3D-printer works, during the printing of the first prototype, a part of the adapter had no support underneath. This resulted in a defect to appear at the upper part of the adapter, at the connector between the handle and the lens cap. Thus, a second prototype was designed. The second prototype (refer to Fig. 13) was overall thicker which directly affects the connector strength. Besides this, the inner surface was also curved which increases the surface area of the connection and improves the 3D-printing process. Furthermore, a ramp was added on top of the lens cap that connects to the connector which also serves to provide better bond between those two parts. After testing, it was found that the second prototype does not provide enough support to hold smartphones in place with the PanOptic ophthalmoscope. Thus, a third prototype was developed with improved structure design.

**Fig. 14:**
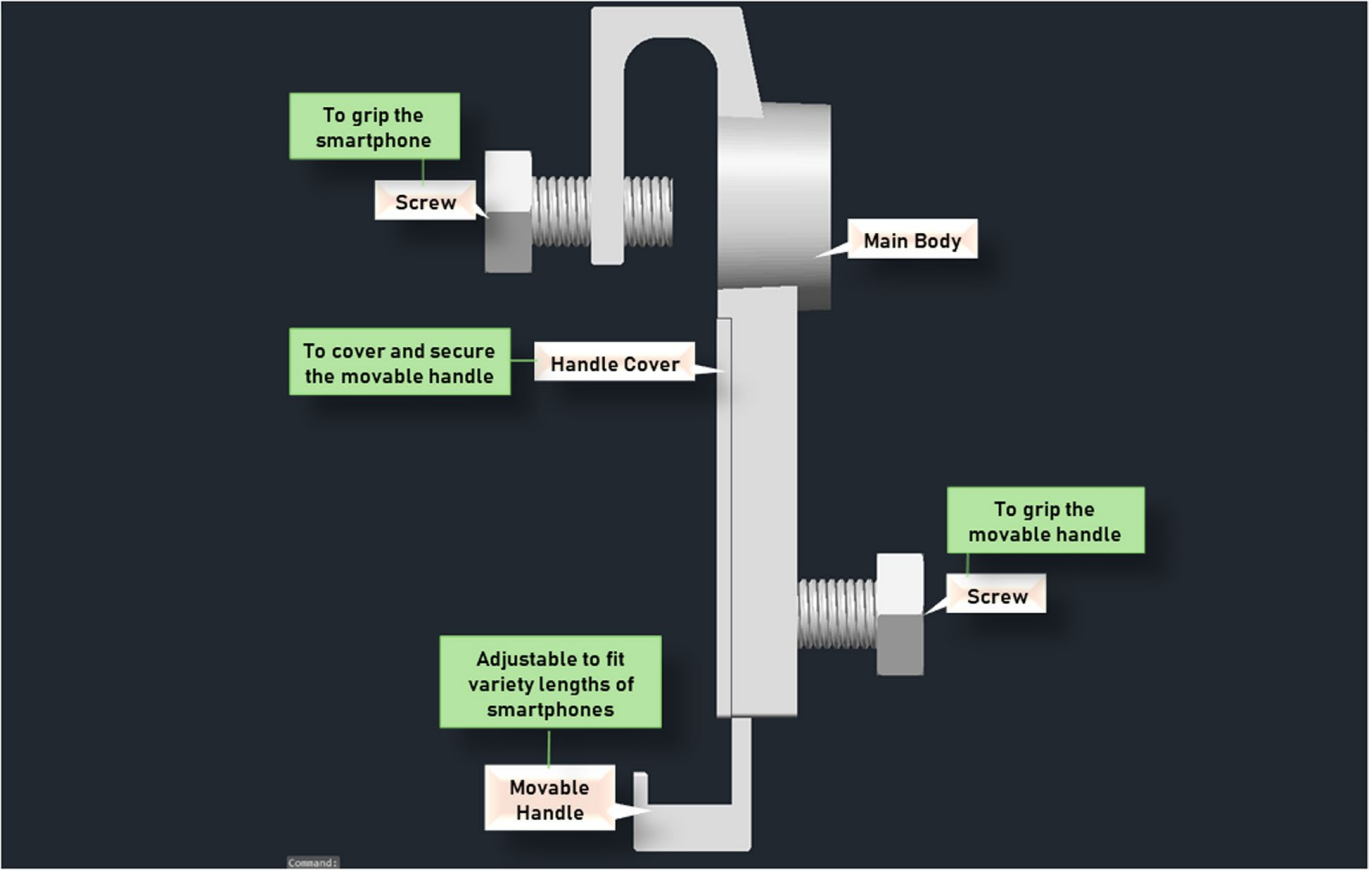
Concept of third prototype in AutoCAD 3D

The third prototype was divided into three main parts which were the main body, the movable handle, and the handle cover (excluding the 3D-printed screws that holds the system altogether). Figure 14 shows the design of the third prototype viewed in AutoCAD 3D. Additional files show the technical drawings for the third prototype [see Additional files 2, 3, and 4]. Referring to Fig. 14, the main body was extended to house the movable handle. The length and width of the main body of the adapter is 150.00 mm and 43.60 mm respectively. The width of the main body was measured as the overall measurement following the dimension of the lens cap. The height of the main body is 51.05 mm. The inner centre of the main body was designed to provide a gap of 4.00 mm to fit the movable handle. The movable handle was made adjustable to fit a variety length of smartphones while providing sufficient support to hold the smartphones where its length, width, and total height is 105.00 mm, 23.00 mm, and 25.0 mm respectively. The handle cover is simply to cover and secure the movable handle where its length, width, and height is 85.00 mm, 30.00 mm, and 3.00 mm respectively.

**Fig. 15:**
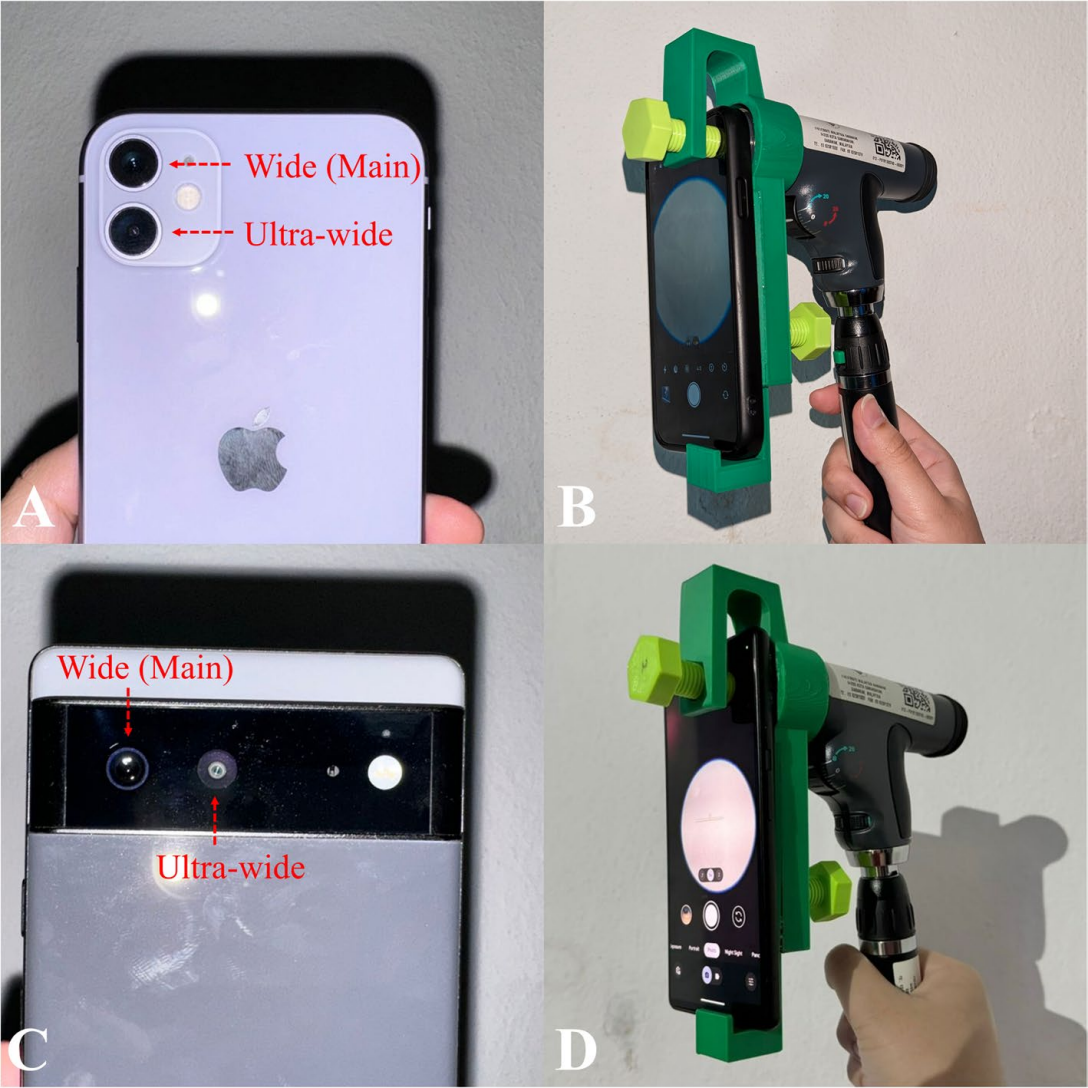
Camera position of iPhone 11 and Google Pixel 6 and assembly with PanOptic ophthalmoscope. **a** iPhone 11 camera position. **b** Assembly of iPhone 11. (**c**) Google Pixel 6 camera position. **d** Assembly of Google Pixel 6

**Fig. 16:**
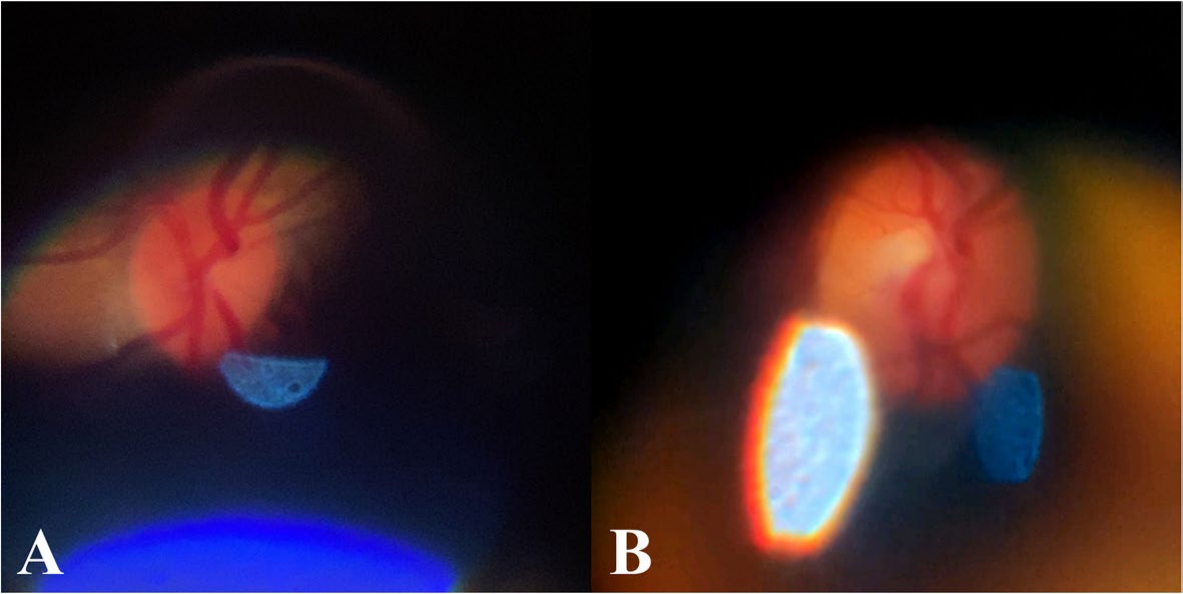
Samples of retina images. **a** Image taken using iPhone 11. **b** Image taken using Google Pixel 6

From the testing, the position of the main camera (wide) lenses for all smartphones had no issues in camera alignment with the PanOptic ophthalmoscope. The ultra-wide camera lenses of the iPhone 11, iPhone 14, and Google Pixel 6 were also tested and showed no alignment issues. Moreover, the movable handle was able to accommodate the different dimensions of the smartphones. Figure 15 shows the comparison of the camera lenses position for the iPhone 11 and Google Pixel 6 together with the assembly when the PanOptic ophthalmoscope is assembled with the iPhone 11 and the Google Pixel 6. As shown in Fig. 15, both smartphones are aligned with the PanOptic ophthalmoscope despite the different dimensions and position of the camera lenses. Figure 16 shows an example of images taken through the iExaminer mobile application using the iPhone 11 and Google Pixel 6 respectively during prototype testing.

**Fig. 17:**
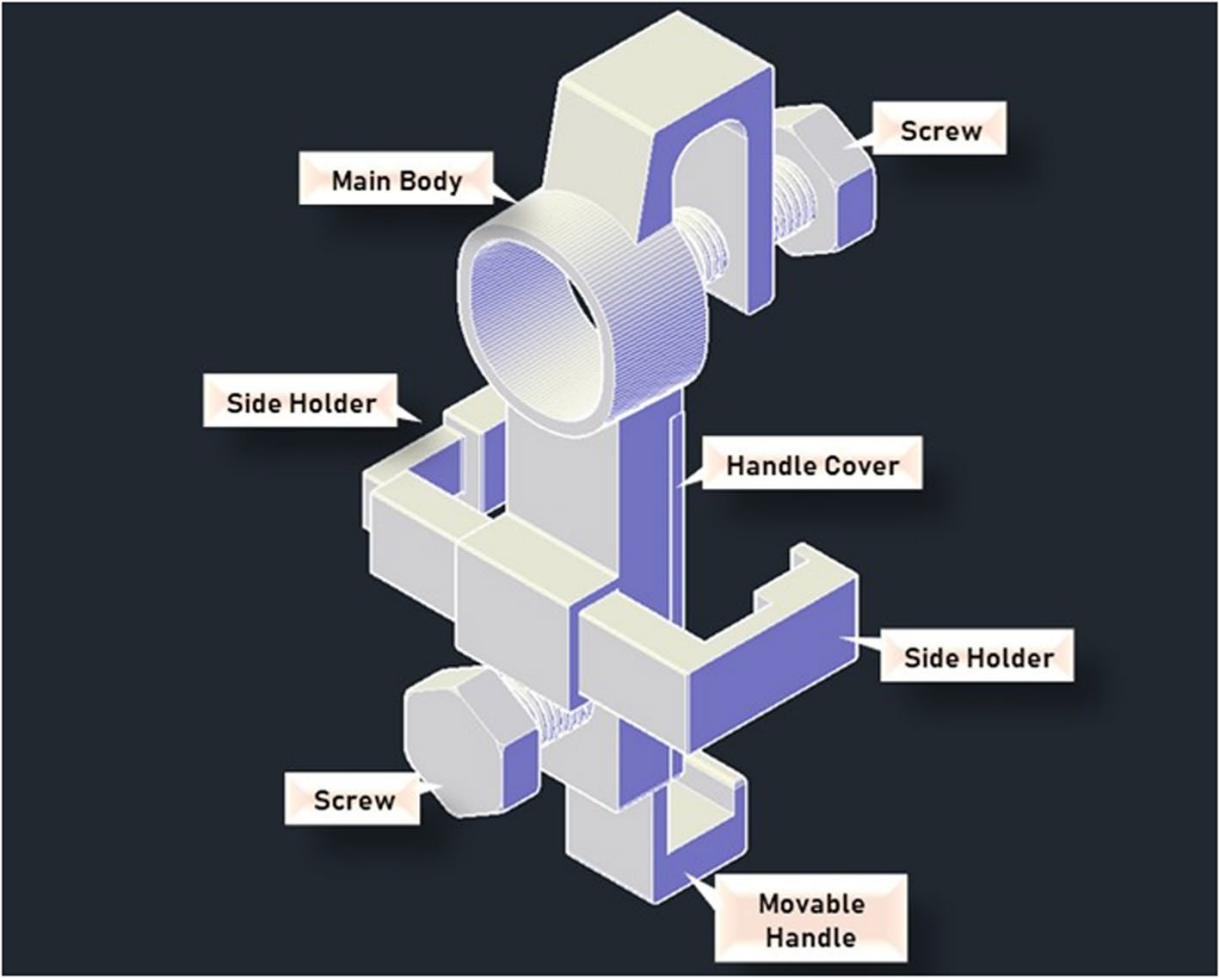
Assembly concept of modified universal adapter in AutoCAD 3D

Furthermore, the third prototype adapter was able to provide sufficient support to hold a smartphone when attached onto the PanOptic. However, the stability and security of the whole assembly raised a concern as there is no support at the sides. This suggests the risk of the smartphone slip-away during retinal imaging. Thus, modifications were made to improve the third prototype which resulted in the final prototype. Figure 17 shows the overall assembly concept of the modified adapter which is also the finalized prototype. The main change of the final prototype is the main body, where a slot was added at the back of the body to insert the side holders which comes in a set of two. The measurement of the rest of the main body remained unchanged where only the overall width of the main body had increased from 43.60 mm to 57.10 mm approximately due to the addition of the slot at the back. Additional files show the technical drawings for the modified main body and side holders [see Additional files 5, 6, and 7].

**Fig. 18:**
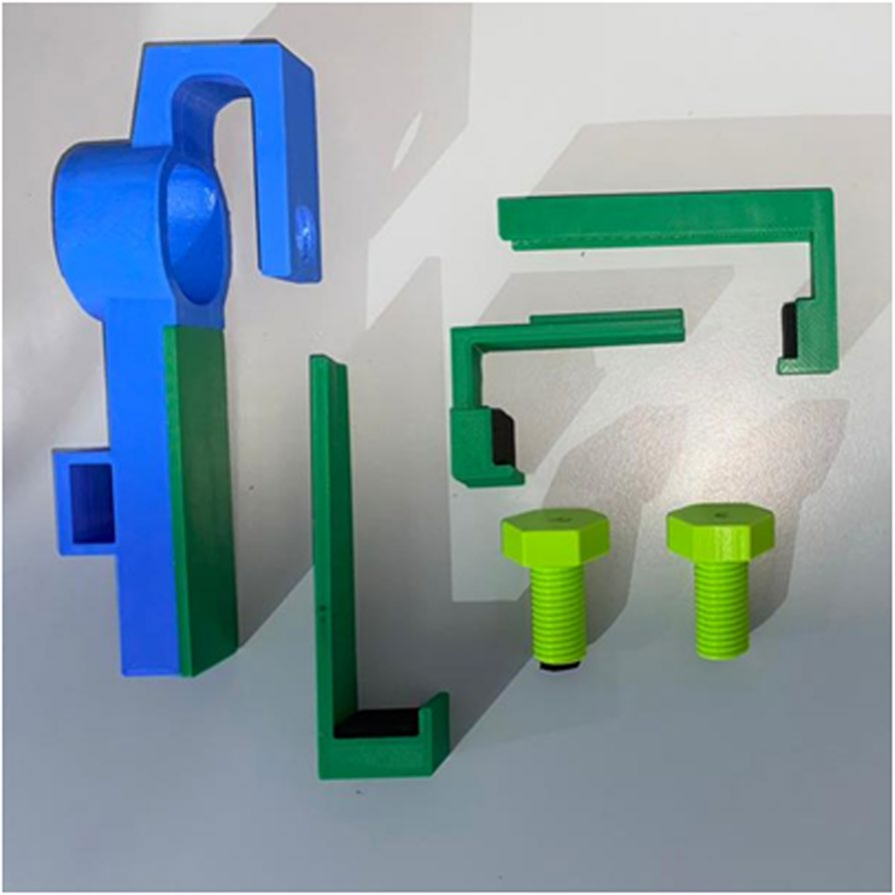
Dissembled 3D-printed modified universal adapter

**Fig. 19:**
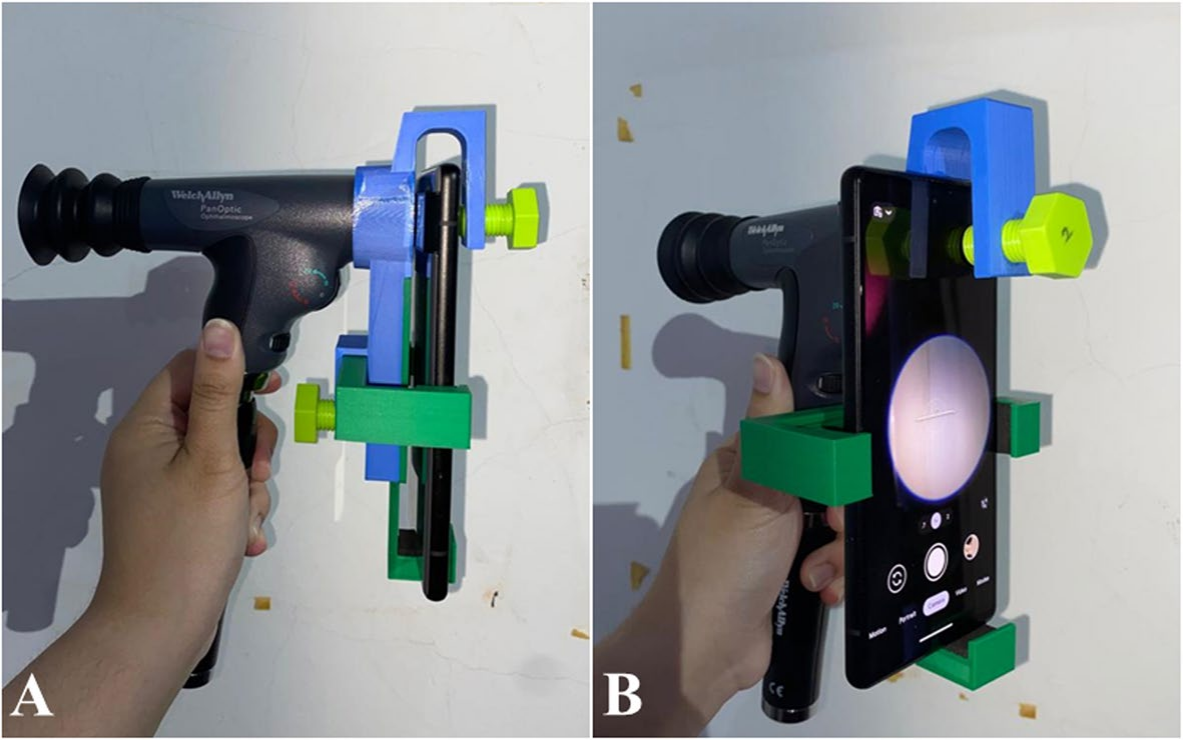
Assembly of finalized universal adapter with PanOptic ophthalmoscope and Google Pixel 6. **a** Sideview. **b** Angled perspective

Figure 18 shows the 3D-printed modified universal adapter (all parts) disassembled which includes the side holders and screws. In addition, grip tapes are added at specific areas which are (i) the inner bottom section of the movable handle, (ii) the inner side area of the two side holders, and (iii) bottom area of the screw that is used to secure the smartphone onto the adapter and PanOptic ophthalmoscope. The grip tape functions to prevent damage onto the smartphone’s screen and for better friction so that the smartphone used would not slide easily. Figure 19 shows the assembly of the modified universal adapter with the PanOptic ophthalmoscope and smartphone of model Google Pixel 6.

### Stress analysis performance

**Fig. 20:**
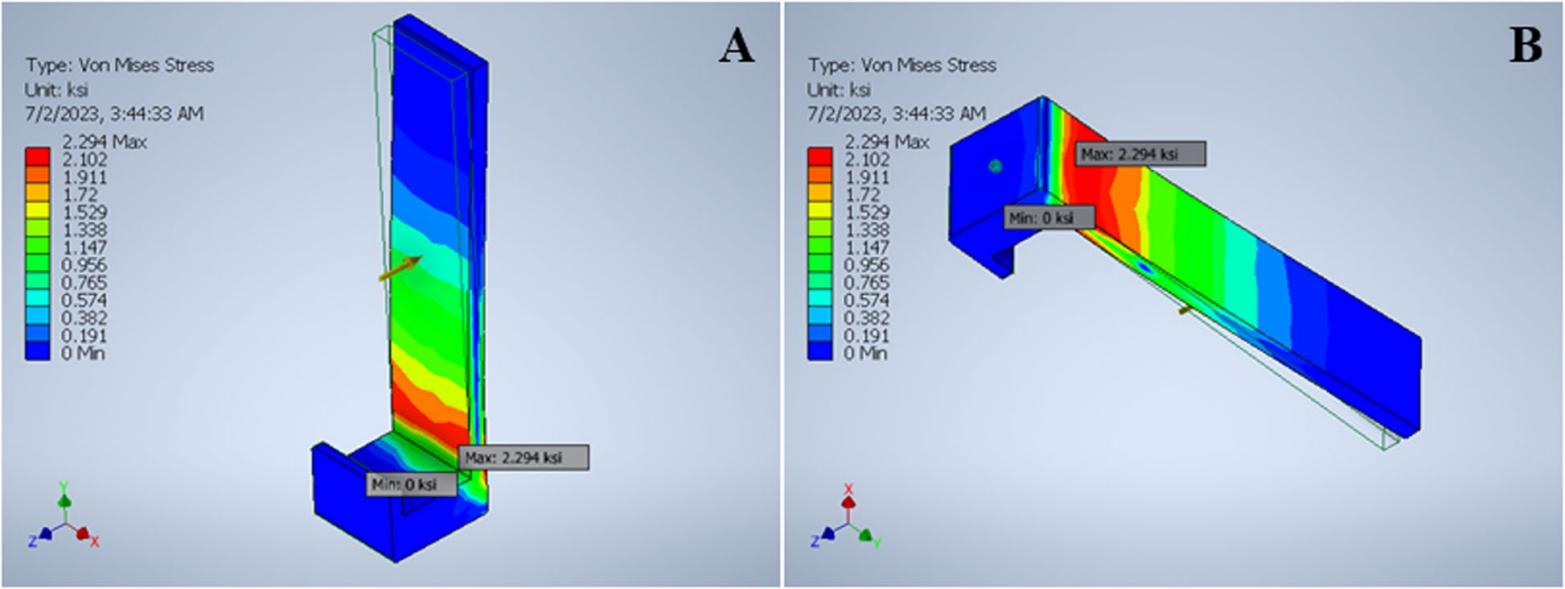
Von Mises stress result of movable handle of adapter. **a** View from the front. **b** View from the back

For the first performance evaluation experimentation which is the stress analysis, Fig. 20 presents the von Mises stress result of the movable handle as an example of the stress result generated using the FEA method in Autodesk Inventor. An outline of the original form of the movable handle before load is applied is represented with thin green lines meanwhile the colored object is a representation of possible deformed appearance of the object. A concentrated red area is observed at the lower part near the base of the movable handle, which indicates the area that has the highest vulnerability and the first area to deform if exceeding the material’s maximum yield strength. Based on Fig. 20, the movable handle experiences a maximum von Mises stress of 2.294 ksi when 5lbf of force is applied. However, the PLA material has a maximum yield strength of 5.816 ksi. Hence, this indicates that the movable handle can hold greater load before it begins to deform permanently as it has not reached the material’s maximum yield strength. It is also noted that the base and the top area of the movable handle experiences the least stress represented by the concentrated blue color. Besides this, the calculated maximum 1st principal stress is 2.290 ksi which is also much lower than PLA’s ultimate tensile strength of 9.123 ksi, hence, the force applied does not compromise the integrity of the model. Table 6 summarizes the minimum and maximum von Mises and 1st principal stress experienced by each part of the final prototype under applied load.

**Table 6:** Stress results of developed universal adapter using FEA method in Autodesk Inventor

Parameters	Movable Handle		Handle Cover		Main Body		Right Holder		Left Holder	
	Min	Max	Min	Max	Min	Max	Min	Max	Min	Max
Von Mises Stress (ksi)	0.000222	2.294	0.00195	3.676	0.0000947	3.869	0.000000302	1.302	0.000000220	2.550
1st Principal Stress (ksi)	-0.147	2.290	-1.608	3.457	-0.690	4.640	-0.663	1.649	-0.289	2.981

### Subjective image quality assessment

**Fig. 21:**
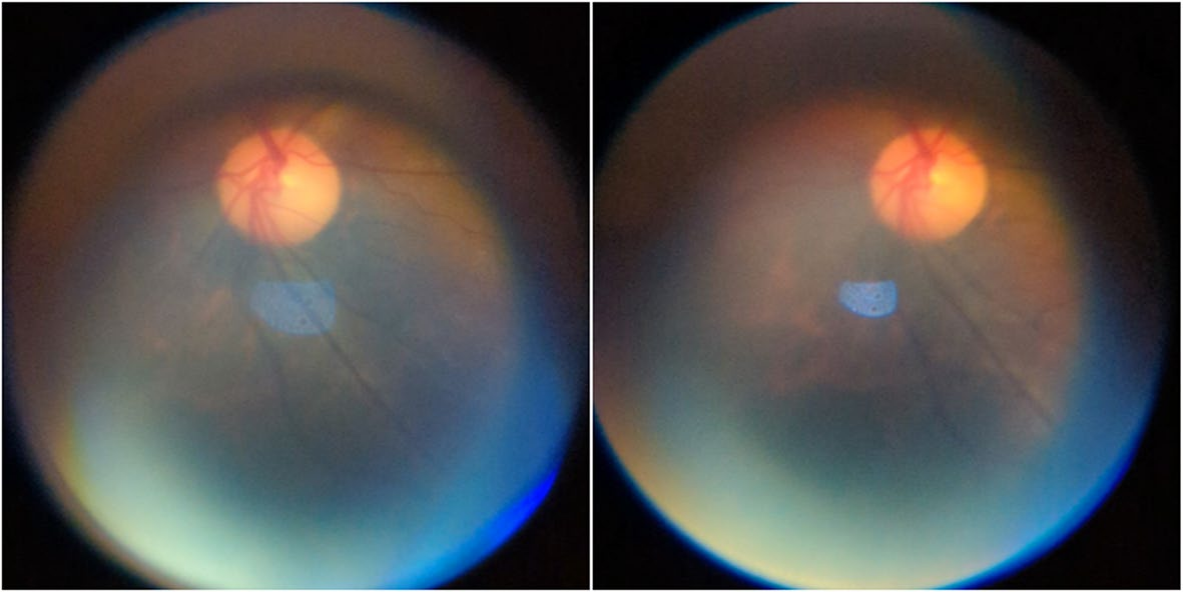
Samples of images acquired by one of the experts

**Fig. 22:**
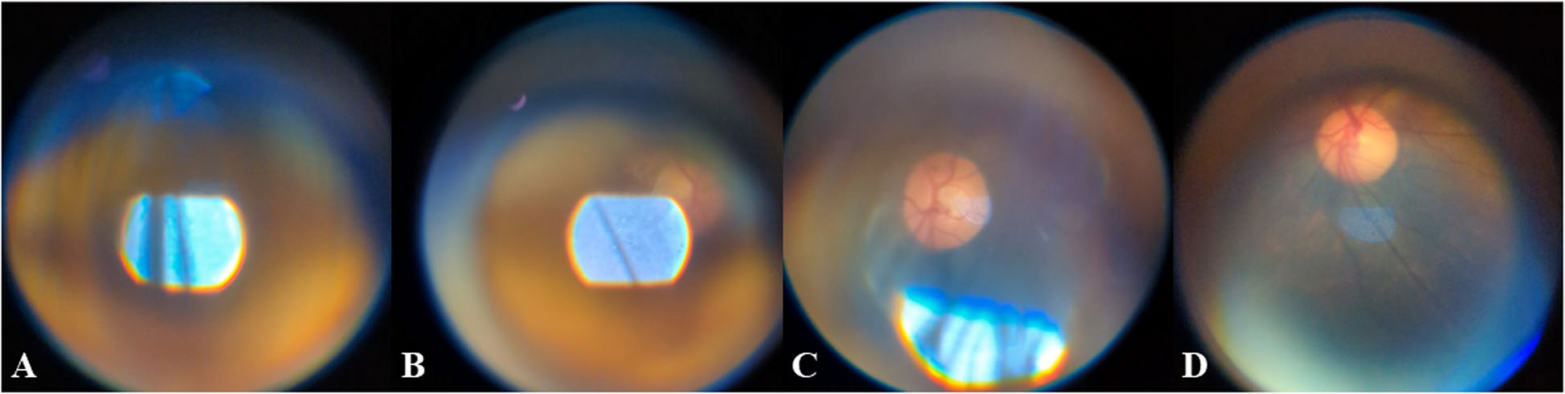
Example of images as graded by one of the experts. **a** Grade 1. **b** Grade 2. **c** Grade 3. **d** Grade 4

For the Subjective Image Quality Assessment, a total of 53 videos were recorded by the experts (examiners) with an average of 4 videos per patient from the clinical testing. Figure 21 shows two examples of the best images obtained by one of the examiners. Referring to Fig. 21, the optic disc and its surroundings can be observed. A total of 140 images were graded by each expert and reviewed. From the review, 37.83% (95% CI = 22.16% to 53.47%) of the images were found to be CA. Figure 22 shows a set of images that have been graded by one of the experts. Referring to Fig. 21, Grade 1 was given to an image where no optic disc was visualized meanwhile Grade 2 was given to an image where the fundus was slightly visualized, and an unclear view of the optic disc. Lastly, Grade 3 was given to an image where there was a clear view of the optic disc and Grade 4 was given to an image with a clear view of the optic disc and its surrounding structures. Grade 5 images, which refer to images that have a clear view of both the optic disc and the macula, were not obtained from the Clinical Testing as graded by the experts.

**Fig. 23:**
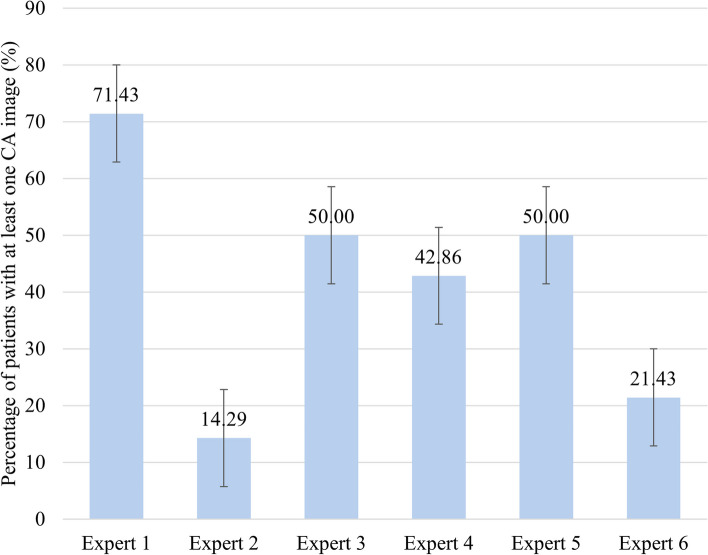
Percentage of patients with at least one CA image graded by each expert

Figure 23 shows the percentage of patients with at least one CA image according to each expert among the 14 patients with standard error bars. Based on Fig. 23, the highest percentage of patients having at least one CA image was observed by Expert 1 which was 71.43% and no overlapping standard error bars were observed for Expert 1 with the rest of the experts. In contrast, a lower percentage was observed for Expert 3, Expert 4, and Expert 5 and have shown small differences among each other which are 50%, 42.86%, and 50% respectively. The standard error bars for these three experts also overlaps which indicates minimal to no difference. A significant difference was observed when comparing the percentage of patients with at least one CA image observed by Expert 3, Expert 4, and Expert 5, with the other three experts. According to Expert 2 and Expert 6, there are 14.29% and 21.43% of patients with at least one CA image respectively. Both Expert 2 And Expert 6 showed overlapping in standard error bars which also indicates minimal to no difference.

**Fig. 24:**
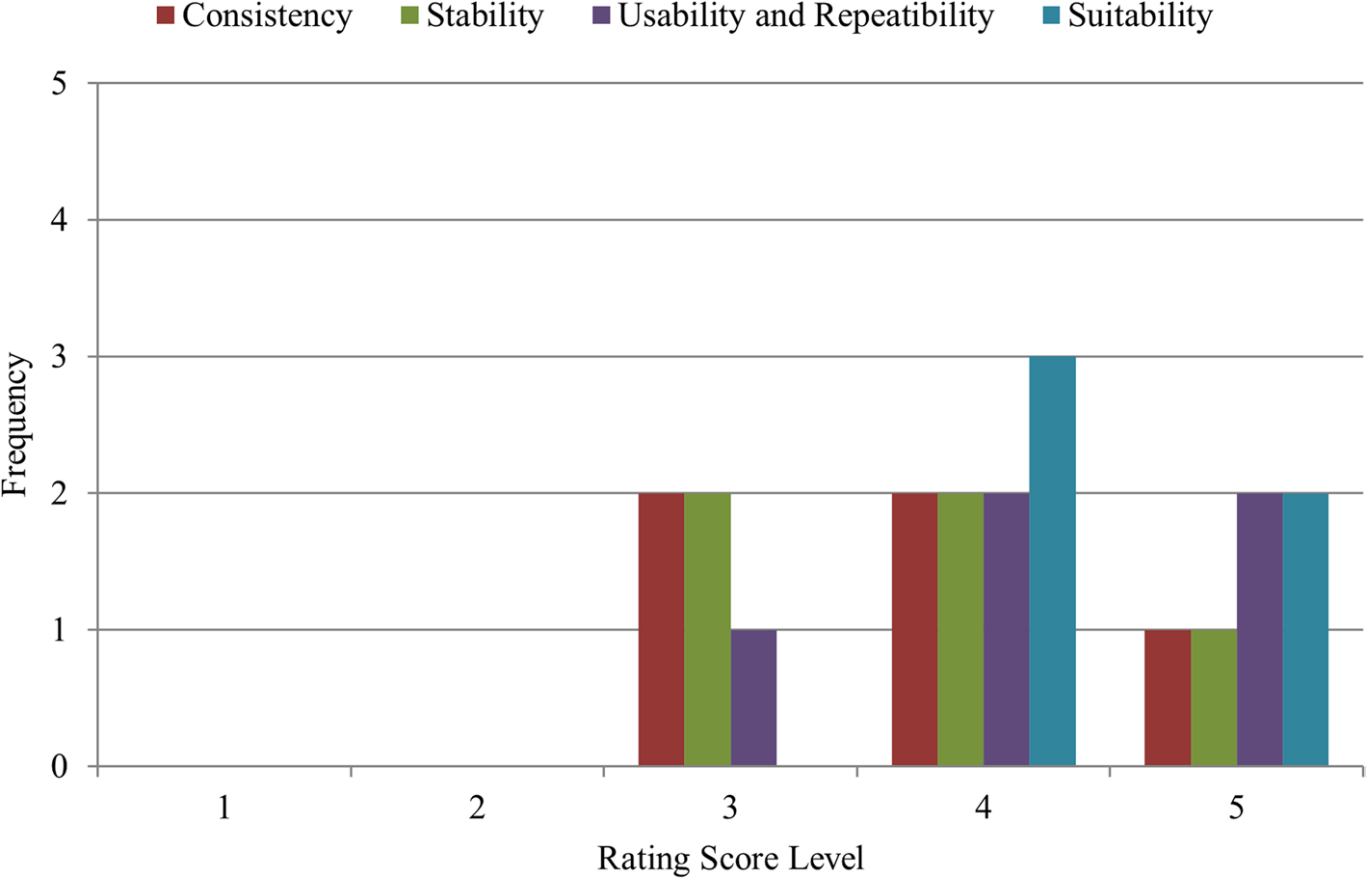
Histogram of the frequency of rating score for the consistency, stability, usability and repeatability, and suitability of the device

Next, the distribution of ratings from the experts on the (i) consistency, (ii) stability, (iii) usability and repeatability, and (iv) suitability of the device are shown in Fig. 24. The x-axis represents the five rating score levels, while the y-axis represents the frequency of experts who selected that specific rating score level. For the consistency, a minimum rating score level of ‘3’ was obtained, meanwhile two examiners provided a rating score level of ‘4’ and the other two examiners provided a rating score of ‘5’. For the stability, a minimum rating score level of ‘3’ was obtained from two examiners, two examiners presented a rating score level of ‘4’, and one presented a rating score level of ‘5’. As for usability and repeatability, positive scores were also obtained. Two examiners gave a score of ‘5’, another two examiners rated it at ‘4’, and the last examiner gave a score of ‘3’. Finally, positive ratings were obtained for the suitability of the developed adapter for the PanOptic ophthalmoscope where two examiners gave a score of ‘5’ and the other three examiners gave a score of ‘4’. Overall, the examiners have given positive ratings on all four aspects. The minimum rating level score given by the examiners was ‘3’ for all four aspects, with the exception on the ratings on the suitability of the overall device which showed the best result where the minimum rating level score given by the examiners was ‘4’.

## Discussion

Reverse engineering has a significant role for the development of proposed adapter. In general, reverse engineering refers to the process of extracting significant information from an existing object and hence the information is utilized to create a new model [43]. According to Helle et al. [44], reverse engineering is employed for a variety of reasons for instance when a product documentation is lost, or the product is no longer in production, the product needs a digital computer-aided design (CAD) model for future modifications or improvements, and more. Thus, this method is useful in developing the proposed universal adapter. In addition, this helps to determine the mechanism of the ophthalmoscope and how the adapter is attached to the ophthalmoscope.

As for the 3D-printing process, it requires constant observation throughout the process to ensure it proceeds smoothly. Furthermore, the selected FDM method ensures sufficient support required for efficient 3D-printing process for the adapter is provided by dividing the printing process into two materials which are the disposable structure and the main part of the model. Referring to Fig. 14, the main body of proposed adapter was designed to have one section for the insertion of a screw at the top to secure smartphones onto the rest of the adapter’s body. The section is connected to the rest of the adapter’s body through a bridge-like structure which results it to be a hanging structure during printing. Hence, the FDM method ensures a disposable structure is printed underneath the hanging structure to acts as a support during the printing process. Disposable structures are also printed underneath the main body of the adapter due to the shape of the lens cap and its size being bigger than the holder of the adapter. Furthermore, pre-heating both the printing bed and filament, and levelling the printing bed is important to avoid damaging the printing bed and printing errors.

From the prototype testing, it has been proven that the developed adapter is universal as it was found usable with the four smartphones that have different dimensions and camera lenses positions. With the lens cap designed to have a large opening, it provides the flexibility to position a smartphone accordingly regardless of the location of the camera lens. The movable handle has also shown its compatibility with the different heights of the four smartphones. In a case where a user has a smartphone with a shorter height, the movable handle can be easily adjusted in AutoCAD 3D to accommodate the required height. In addition, the images obtained using the iPhone 11 and Google Pixel 6 showed a relatively small field of view of the retina, but the optic disc and blood vessels can still be observed as shown in Fig. 16. From the prototype testing, it was difficult to obtain good quality retina images and the duration taken to familiarize with the Pan Optic ophthalmoscope was quite long. Furthermore, glares and reflection were also visible on the images obtained. However, the process of viewing the retina was easier when the Google Pixel 6 was used. The Google Pixel 6 was able to focus onto the optic disc notably faster when compared to the iPhone 11. Furthermore, based on Fig. 16, it can be observed that the Google Pixel 6 captured more details of the optic disc when compared to the images captured using the iPhone 11. This is because the Google Pixel 6 features a camera resolution of 50 megapixels meanwhile the iPhone 11 only provides 12 megapixels. Meanwhile, the iPhone 6 and iPhone 6 s/ 6 s plus (the only smartphone series that is compatible with the PanOptic’s original adapter) only provides 8 megapixels and 12 megapixels respectively. This further highlights the importance of developing this universal adapter that allows studies to utilize the latest smartphones technologies.

Besides this, results obtained from the stress test were positive. The overall calculated maximum von Mises stress and 1st principal stress calculated for each part of the final prototype from the stress test conducted were all lower than PLA’s maximum yield strength and ultimate tensile strength (refer to Tables 4 and 6). Thus, this shows that the design and material of all three parts are sturdy and is unlikely to deform under applied force. Several studies have also developed their own smartphone-based 3D adapters where the adapters are utilized directly with a pair of lenses [20–22]. However, there is a lack in describing the integrity and longevity of the adapters. Besides the performance of the 3D-printed adapters in retinal imaging, it is also important to conduct stress tests on the adapters that ensure replicability or reproducibility in case of high demand. Furthermore, understanding the physical and mechanical properties when developing a prototype helps to prevent any wastage especially when considering that one of the main purposes of the development of smartphone-based retinal image acquisition is to provide an affordable and portable method for patients in low- and middle- income populations that are less likely exposed to early detection of diseases, Hence, the FEA method presented allows direct comparison with the maximum yield strength of the PLA material. This method helps to determine the stress value for proposed adapter that would cause it to begin deforming permanently. In addition, the FEA method determines which areas of proposed parts of the adapter experience the most stress, and areas that are the most sturdy and flexible.

From the Subjective Image Quality Assessment, the images collected by the experts from the clinical testing showed the best image quality as shown in Fig. 21. Based on Fig. 21, the optic disc can be observed clearly, and the images showed a wider field of view, and minimal glares and reflections were observed. When compared with the images collected during the prototype testing, the images collected from the clinical testing are of higher quality. This suggests that there is a significant learning curve in smartphone-based retinal imaging.

Referring to Fig. 23, the highest percentage of patients having at least one CA image observed by Expert (71.43%) indicates that most of the patients had at least one CA image according to Expert 1. However, there is a significant difference between the percentage of patients with at least one CA image as observed by Expert 1 and the rest of the experts. The low percentage obtained from Expert 3 (50%), Expert 4 (42.86%), and Expert 5 (50%) indicates that a low number of CA images were obtained. However, the small differences among Expert 3, Expert 4, and Expert 5 shows a high degree of agreement among them. However, there is a significant difference between the percentage of patients with at least one CA image as observed by Expert 2 and Expert 6 and the other three experts. Based on Fig. 23, the percentage of patients with at least one CA image as observed by each expert can be further grouped into three groups. Group 1 consists of only Expert 1 where the percentage indicates that most patient had at least one CA image which also indicates that most of the images were good quality. Group 2 consists of three experts which are Expert 3, Expert 4, and Expert 5 with an average of 47.62%. This indicates that according to these three experts, a fair number of the images obtained during the clinical testing were clinically adequate. For Group 3, the lowest percentage of patients with at least one CA image was observed by Expert 2 and Expert 6 which were 14.29% and 21.43% respectively. This indicates that according to these two experts, the images obtained from the clinical testing were mostly not clinically adequate.

The results from the end-user survey have shown an overall positive score on all four aspects (consistency, stability, usability and repeatability, and suitability of adapter) as shown in Fig. 24. For the consistency, all examiners agree that the developed adapter when used together with the PanOptic ophthalmoscope is able to produce image of similar quality over time. When it comes to the stability of the assembled device throughout the process, the results also show a similar positive output. Based on the positive scores, it was concluded that there was no issue throughout the retinal imaging acquisition process in terms of stability. Furthermore, the positive score on the stability also suggests that the integrated side holders and grip tapes and overall design of developed adapter is capable to provide optimum stability to the examiners. Experts have also given positive ratings on the usability and repeatability which concludes that the examiners find that the overall adapter is user-friendly, and capable of producing consistent results. The positive ratings received on the suitability also indicates that all examiners agree that the developed adapter is suitable and compatible with the PanOptic ophthalmoscope. However, there is a significant contrast between the positive sentiment towards the performance of the developed adapter from the end-user survey and the overall percentage of CA images from the Subjective Image Quality Assessment. This suggests that the developed universal adapter does not have a significant role in the quality of images taken using the PanOptic ophthalmoscope, but rather the ophthalmoscope itself may not be suitable for non-mydriatic retinal imaging, especially due to its a small field of view of 25° that also directly affects the learning curve of the device.

## Conclusion

In conclusion, the concept of proposed adapter was presented in this paper with the objective of creating a universal and suitable adapter for retinal imaging using the PanOptic ophthalmoscope. The developed universal adapter in this paper was tested with four smartphones that have different camera position and dimensions. All four smartphones have shown no issues in camera alignment with the PanOptic ophthalmoscope. In this research, two experiments were conducted for the performance evaluation on the developed adapter which is the finite element analysis (FEA) method for the stress test, and Subjective Image Quality Assessment involving a non-mydriatic clinical testing and an end-user survey for the second experiment. From the stress test, it was found that the developed adapter is sturdy and durable since the calculated von Mises and 1st principal stress are far below PLA’s yield and ultimate tensile strength. The contrasting results from the Subjective Image Quality Assessment and end-user survey suggest that the overall performance of the developed adapter is good and reflects more on the suitability and efficiency of the PanOptic ophthalmoscope for non-mydriatic retinal imaging. The main advantage of this research is the developed adapter being a product of 3D-printing which is low-cost, easily reproducible and modified for improvements. One of the main limitations of this research is the experimentation procedures for the retinal imaging may be insufficient in validating its usability and performance overall. The clinical testing conducted also involved a small number of patients and experts which may not be sufficient to define the performance of the developed adapter. Besides this, the design of the proposed adapter itself has its own limitations in terms of stability and security in providing an efficient retinal imaging process. Thus, for future work, the design can be further improved and analyzed that reflects its usage in clinical practices. In addition, other methods of stress analysis such as experimental stress analysis or topology optimization should be employed to validate the performance of the proposed adapter. Experimental stress analysis method is a method that involves physically testing the proposed adapter with various loading conditions meanwhile the later method uses computer algorithms for the optimization of proposed adapter given a set of loading condition. Other than that, more types of low-cost materials such as acrylonitrile butadiene styrene (ABS) and polyethylene terephthalate glycol-modified (PETD) with higher yield strength should be explored as an alternative in developing the universal adapter especially if the proposed adapter is to be made commercial in the future.

### Supplementary Information

Additional file 1: Technical drawing of screw.

Additional file 2: Technical drawing of main body.

Additional file 3: Technical drawing of handle cover.

Additional file 4: Technical drawing of movable handle.

Additional file 5: Technical drawing of modified main body (final prototype).

Additional file 6: Technical drawing of left holder.

Additional file 7: Technical drawing of right holder.

## Data Availability

No datasets were generated or analysed during the current study.

## Abbreviations

ABS: Acrylonitrile butadiene styrene
CA: Clinically adequate
CAD: Computer-aided design
DR: Diabetic retinopathy
FDM: Fused deposition modeling
FEA: Finite element analysis
PDA: Personal digital assistant
PETD: Polyethylene terephthalate glycol-modified
PLA: Polylactic acid
RAM: Random access memory
