# Supplementary figures and images for: Development of 3D-printed universal adapter in enhancing retinal imaging accessibility
Source: 3D Print Med. 2024 Jul 19;10:23. doi: 10.1186/s41205-024-00231-0 (PMC11264814; doi:10.1186/s41205-024-00231-0)

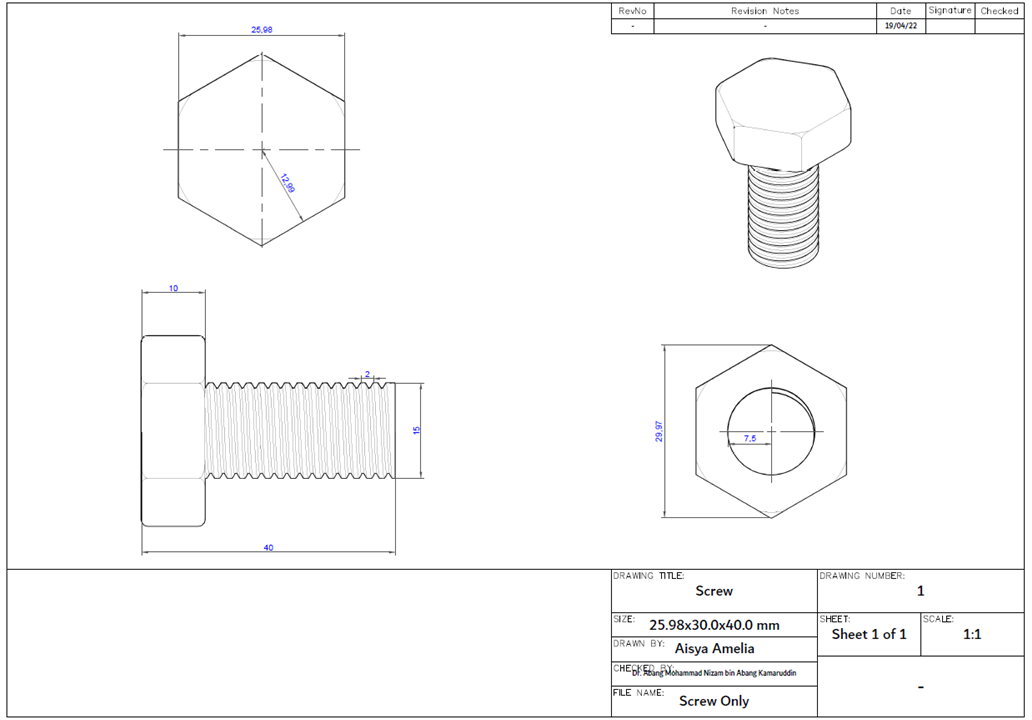

Supplement: Supplementary file 1 — Additional file 1: Technical drawing of screw. [file 41205_2024_231_MOESM1_ESM.png]

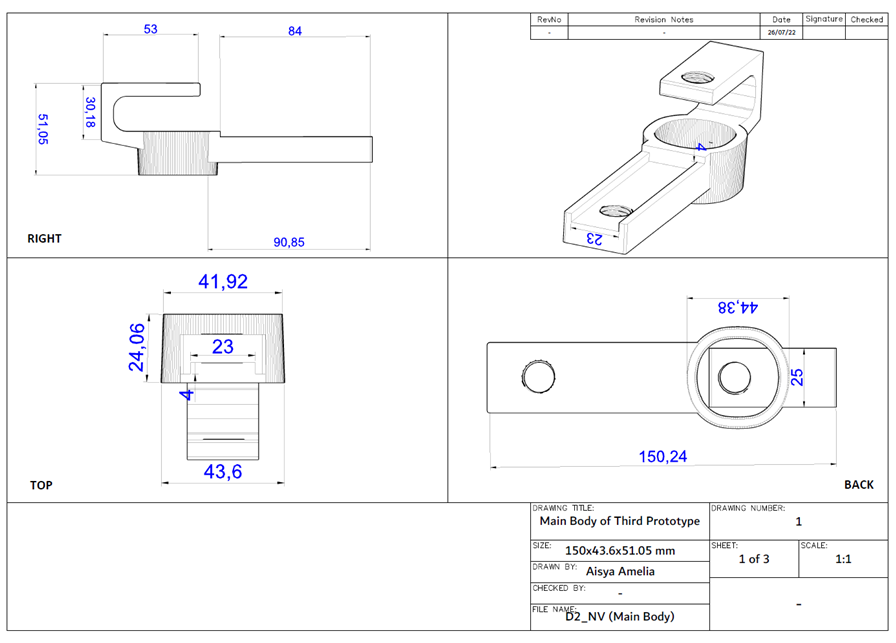

Supplement: Supplementary file 2 — Additional file 2: Technical drawing of main body. [file 41205_2024_231_MOESM2_ESM.png]

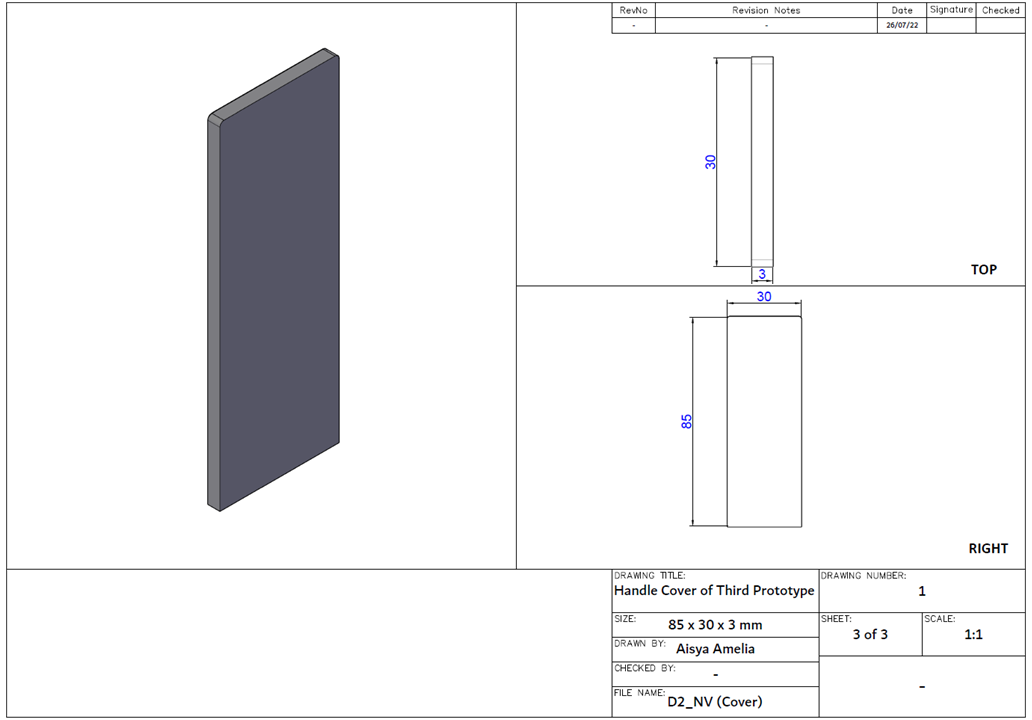

Supplement: Supplementary file 3 — Additional file 3: Technical drawing of handle cover. [file 41205_2024_231_MOESM3_ESM.png]

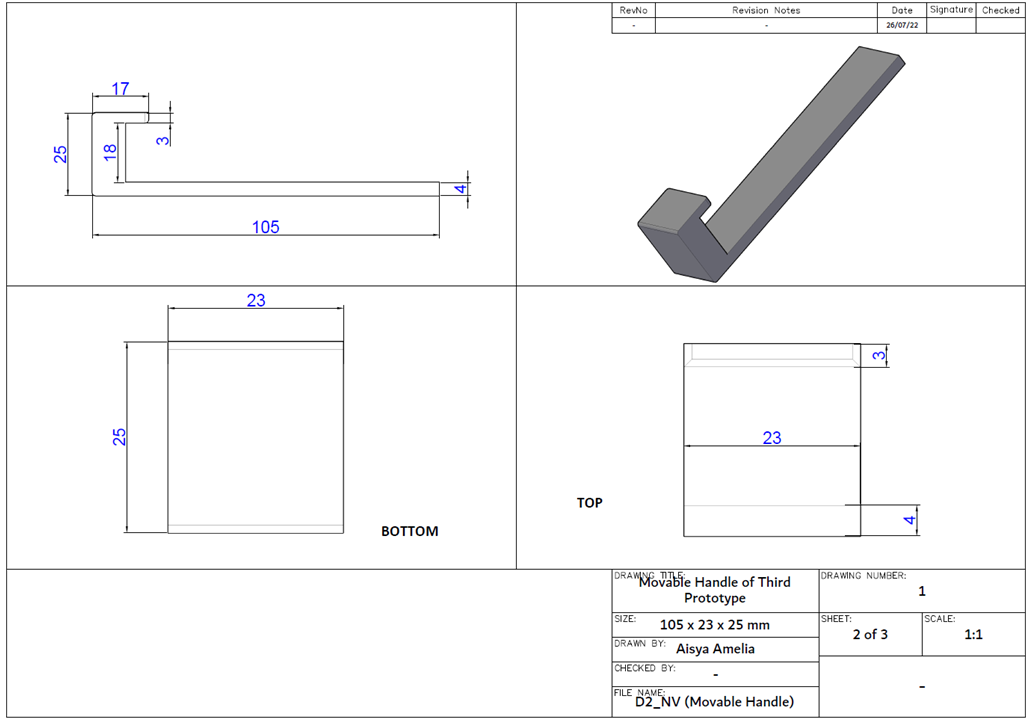

Supplement: Supplementary file 4 — Additional file 4: Technical drawing of movable handle. [file 41205_2024_231_MOESM4_ESM.png]

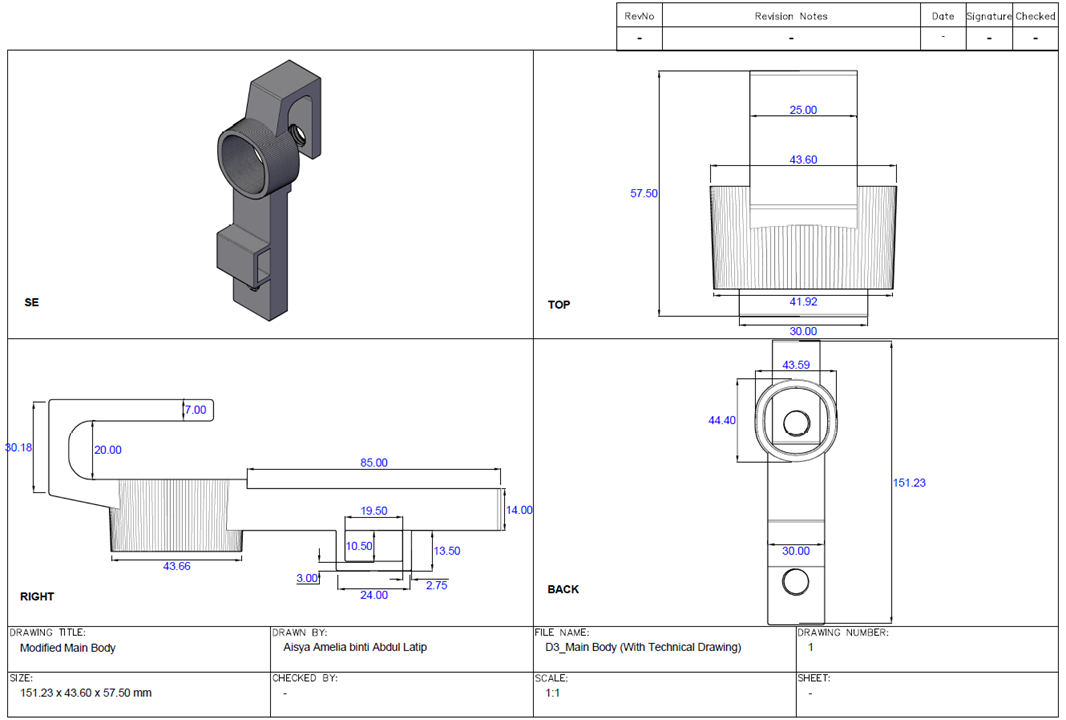

Supplement: Supplementary file 5 — Additional file 5: Technical drawing of modified main body (final prototype). [file 41205_2024_231_MOESM5_ESM.png]

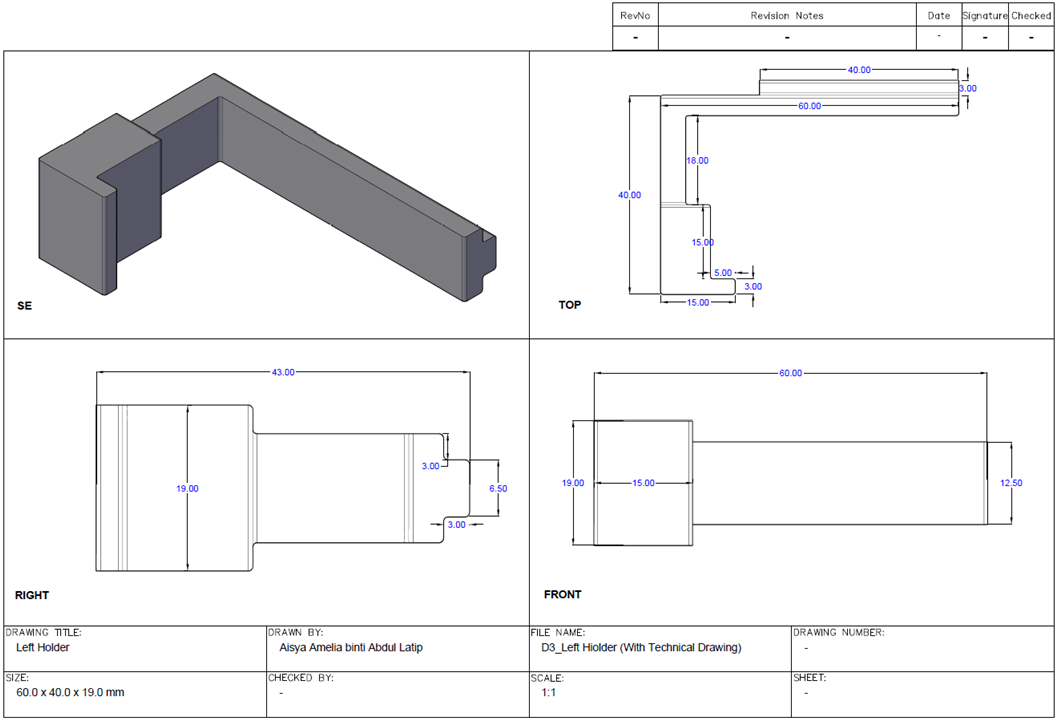

Supplement: Supplementary file 6 — Additional file 6: Technical drawing of left holder. [file 41205_2024_231_MOESM6_ESM.png]

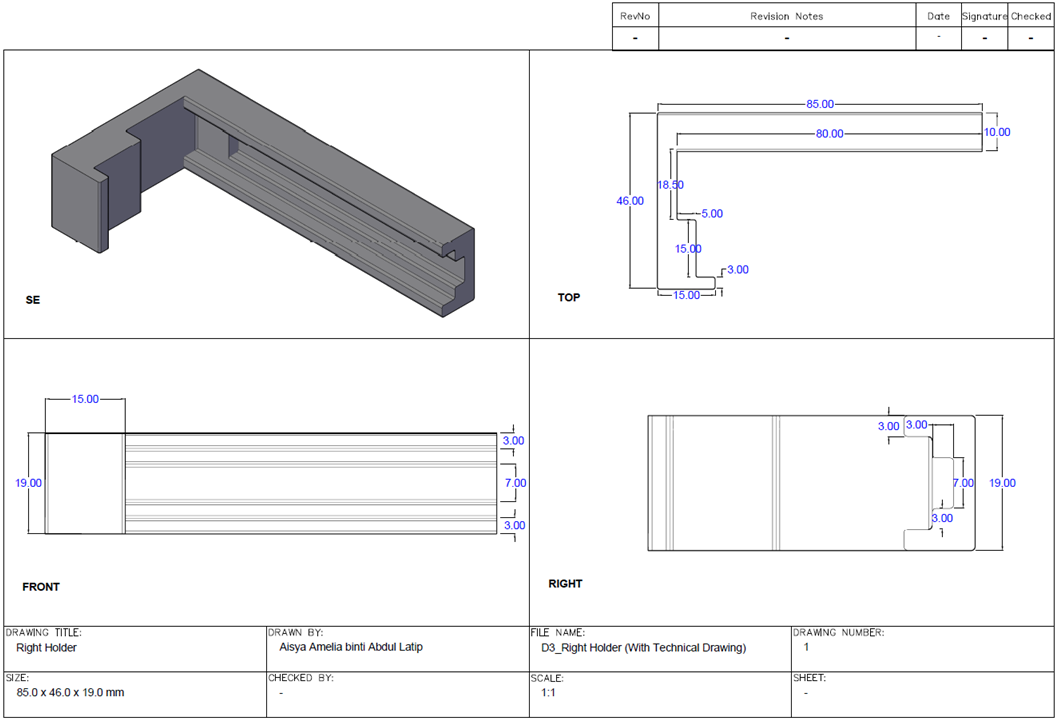

Supplement: Supplementary file 7 — Additional file 7: Technical drawing of right holder. [file 41205_2024_231_MOESM7_ESM.png]
